# Optimizing emissions and carbon credit from integrated solid waste and wastewater management: A MATLAB-based model with a Graphical User Interface (v1)

**DOI:** 10.1016/j.mex.2020.100839

**Published:** 2020-02-24

**Authors:** Amani Maalouf, Mutasem El-Fadel

**Affiliations:** Department of Civil and Environmental Engineering, American University of Beirut, Lebanon

**Keywords:** SWW, Solid Waste Management, Wastewater Management, Life Cycle Emissions Accounting, Optimization, Economic analysis, Sensitivity analysis, Carbon credit, Decision support, CH_4_, Methane, CO_2_, Carbon dioxide, EFs, Emission factors, FWD, Food waste disposer, GHG, Greenhouse gas, GUI, Graphical User Interface, GWP, Global warming potential, IPCC, Intergovernmental panel on climate change, LCA, Life cycle assessment, LFG, Landfill gas, MSW, Municipal solid waste, MTCO_2_E, Metric tonnes of CO_2_ equivalent, N_2_O, Nitrous oxide, NDCs, Nationally Determined Contributions, OAT, One-at-a-time analysis, SM, Sludge management, SWW, Solid Waste and Wastewater management software, UNFCCC, United Nations framework convention on climate change, WW, Wastewater management

## Abstract

A new Solid Waste and Wastewater (SWW) management software is presented for optimizing the life-cycle of emissions with carbon credit cost considerations. The software is the first to combine integrated solid waste and wastewater management systems under a single framework when introducing a food waste disposer (FWD) policy. The model/software offers a platform encompassing several tools for life cycle emissions accounting, optimization, as well as economic, policy, and sensitivity analysis. It provides the flexibility of selecting processes or modifying input parameters, as well as disaggregating emissions depending on the scope of accounting. The graphical user interface is applicable in the context of developed and developing economies with the ultimate objective to assist decision makers to allocate expenditures for emissions mitigation measures.

## Highlights

• A MATLAB-based graphical user interface allows users to control the operation of the system.
• A user-friendly tool available upon request.
• Successfully tested in the context of developed and developing economies.

## Graphical abstract

**Figure:**
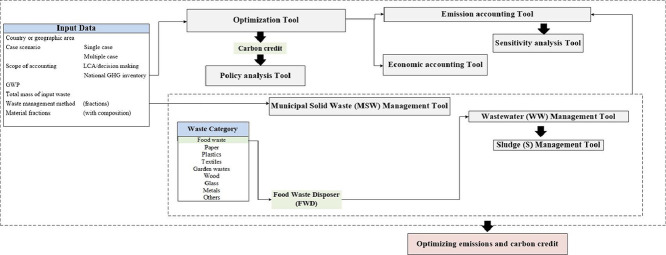

**Table:** Specification Table

Subject Area:	*Environmental Science*
Method name	*Solid Waste and Wastewater (SWW) management software*
More specific subject area:	*Solid waste & wastewater management*
Name and reference of original method:	*Maalouf, A., El-Fadel, M. (2020). A novel software for optimizing emissions and carbon credit from solid waste and wastewater management. Sci. Total Environ., 714, 136736*[1]
Resource availability:	*SWW 1.0 (software available upon request)*

## Method details

Maalouf and El-Fadel [1] presented a review of waste models, tools, protocols, and guidelines commonly reported for emissions accounting, which evolved since the 1970s, showing that all models targeted developed economies with default input data introduced for specific locations and often with uncertainty about emission factors that are not readily accessible or adjustable. Moreover, the review showed that none of the existing emissions' accounting models considered the assessment or policy evaluation of combined solid waste and wastewater management systems when introducing a food waste disposer (FWD) at the household level. This highlights the need for an integrated tool that assists practitioners and decision makers in examining waste management processes within a wider context, with applicability in both developed and developing economies.

The software is based on a life cycle inventory of emissions with several tools for technical, economic, and policy analysis. It also offers an optimization tool based on minimizing total emissions or costs of integrated solid waste and wastewater management systems while considering carbon credit from both options. It provides the advantages of in-depth disaggregation of emissions by source (Food Waste Disposer, collection, recycling, composting, Anaerobic Digestion, incineration, landfilling, open dumping, and open burning), type (direct or indirect), or main gas (CO_2_, CH_4_, and N_2_O). In addition, it includes a built-in Monte Carlo simulation to check on the variability in emissions by varying key parameters.

The software was designed under a Matlab-based Graphical User Interface (GUI) and strengthened with a user- flexibility to select processes or modify input parameters. Matlab is universally accepted as one of the most powerful data processing platforms. Its connectivity with many advanced programming languages (like C, Java, and VB) and availability of a wide range of toolboxes makes it popular among the scientific and research community. The software development can be divided into two phases: (1) hidden programming for data collection and model formulation based on Matlab code, and (2) interface initialization built and executed over the Matlab code using GUI tools. The interface allows the user to select data and input parameters as well as visualize outputs by displaying various forms of plots. The Matlab-based software provides an efficient way to operate and manipulate the data and automatically store results in excel files.

The model provides flexibility in editing the graphs and figures and updating/customizing the databases such as databases for scenario definitions, scope of accounting, GHG inventory, global warming potentials, cost and savings with change in global economy, which are all further described in the below sections.

**Fig. 1:**
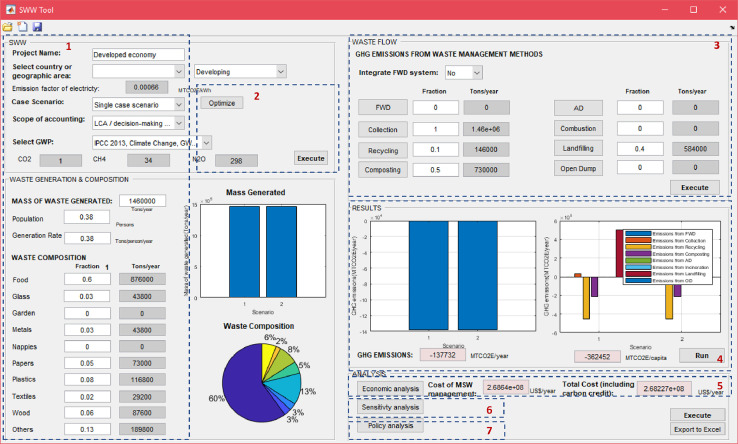
SWW user interface (startup screen). 1: Data input; 2: Optimization tool; 3: Emission accounting tool; 4: Results display; 5: Economic analysis tool; 6: Sensitivity analysis tool; 7: Policy analysis tool.

Fig. 1 depicts the SWW software at the starting mode. This screen shows the user input data as well as the available tools in the software that is described below in more details.

## Input data

When lacking, input data consist of default averages or modifiable by the user through a graphical interface as elaborated below.

### Country or geographic area

**Fig. 2:**
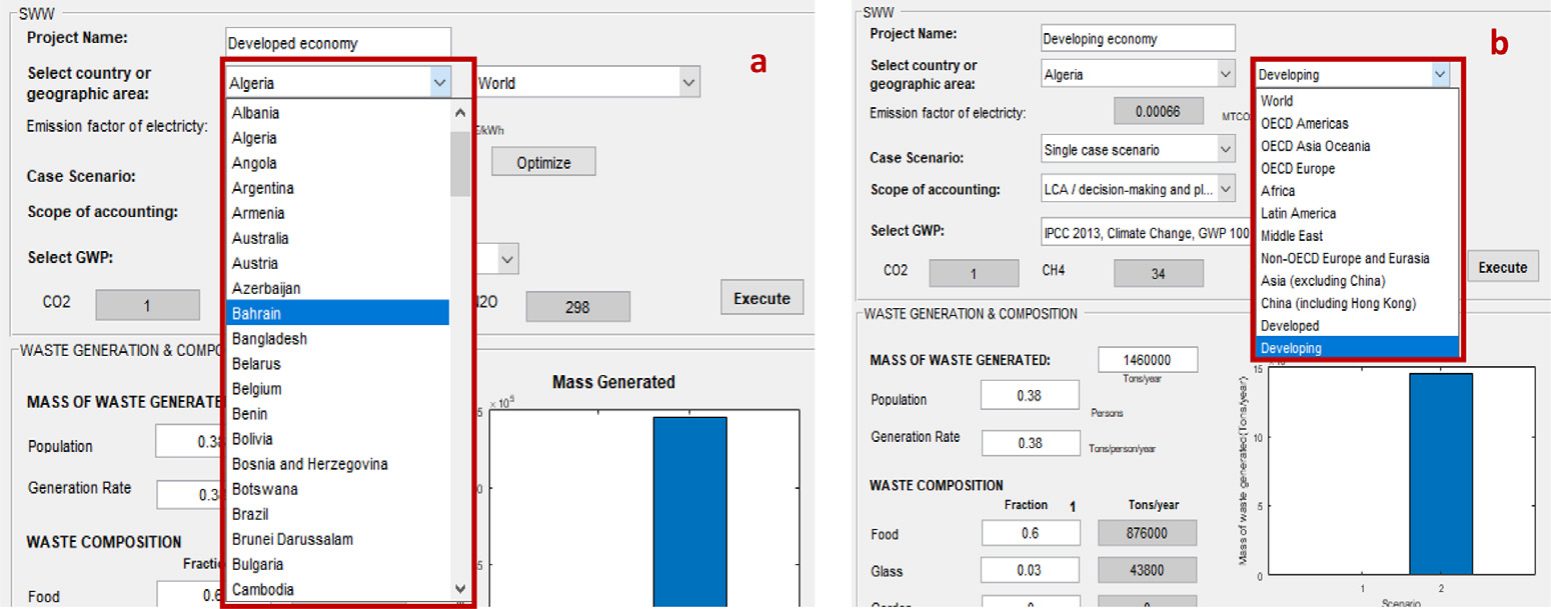
Country or geographic area.

Some data such as the electricity generation mix (e.g. the share of coal, fuel oil, natural gas, nuclear, and renewable electricity generation) are related to geographical conditions. Accordingly, it is imperative for the user to provide location-specific data to ensure representative results. When data is not available, the SWW software offers average default data for emission factors (EFs) of electricity depending on the selected country (Fig. 2(a)) or geographic area (Fig. 2(b)). The electricity data are adapted from the International Energy Agency [2]. First, select the country of study in order to display the average emission factor. In case the user did not specify the country, leave as empty and select the geographic study area. The emission factor will be displayed after clicking on the “execute” button.

### Scenario definition

**Fig. 3:**
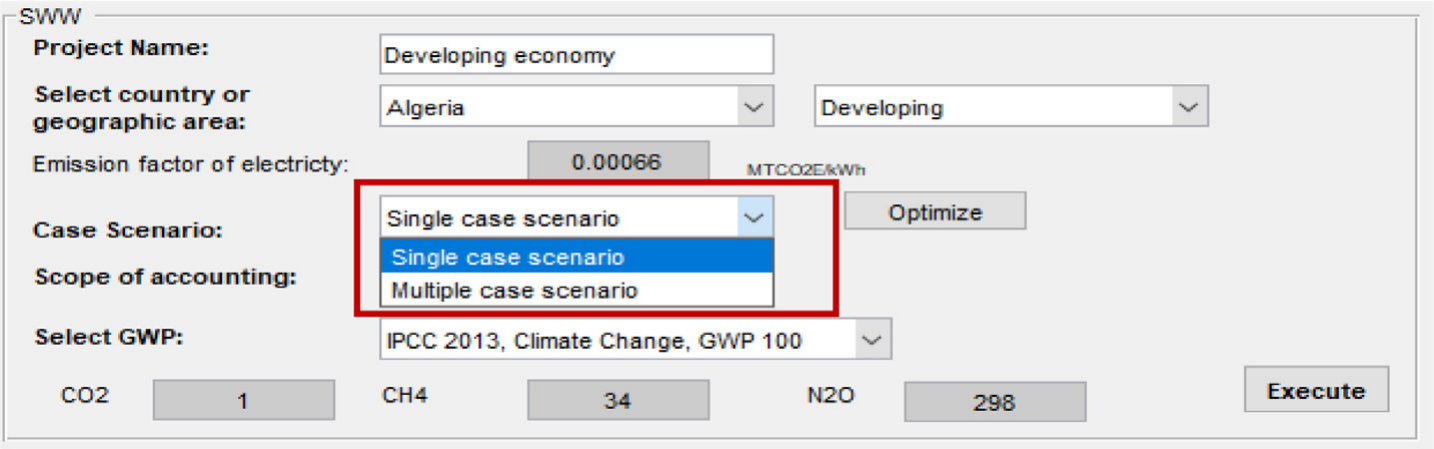
Select case scenario.

The user has the option to select whether to conduct: (1) a “single case scenario” that considers emissions’ estimation from a predetermined waste management system; or (2) a “multiple case scenario” that considers a wide range of possible combinations to optimize the integrated solid waste and wastewater management system based on minimum emissions or costs (see Optimization tool section) (Fig. 3).

### Scope of accounting

**Fig. 4:**
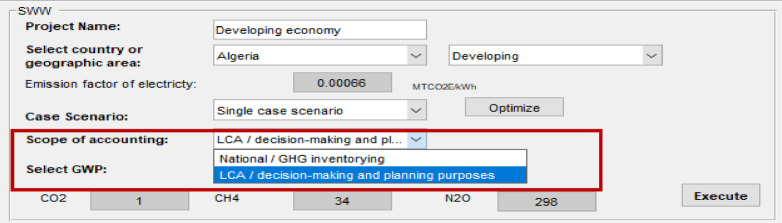
Select scope of accounting.

The software disaggregates emissions by type (direct or indirect), which allows the user to select the scope of reporting whether for “national greenhouse gas (GHG) inventory” (accounting for direct emissions) or “life cycle assessment LCA/planning and decision-making purposes” (accounting for direct and indirect emissions) (Fig. 4). Therefore, the results of total emissions are displayed in the main window (Fig. 1) according to the selected scope of accounting.

### GWP

The global warming potential (GWP) comprises a GWP_20_, GWP_100_ and GWP_500_, for a time horizon of 20, 100 and 500 years, respectively [3]. All reporting mechanisms use GWP values provided by the Intergovernmental Panel on Climate Change (IPCC) based on the effects of GHGs over a 100-year time horizon (GWP_100_). The latter has evolved three times since the Second Assessment Report (SAR) published by the IPCC [4] until the last one (Fifth Assessment Report-AR5) published in 2013 due to improvements in calculations and an increase in atmospheric GHGs during this period.

**Fig. 5:**
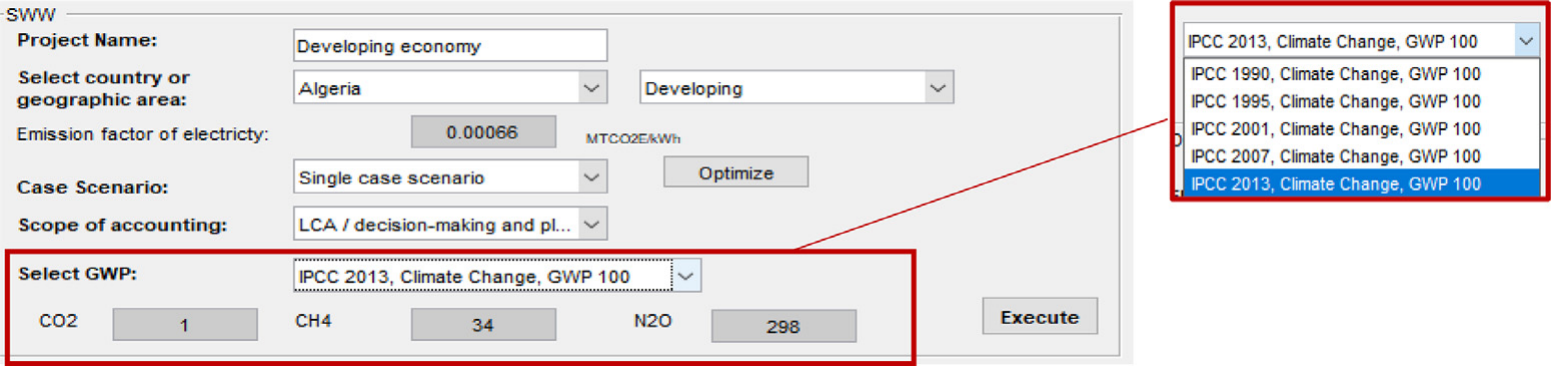
Select GWP.

Regardless of the scope of reporting, the time horizon (e.g. 20, 100, 500 years) must be defined and the reference of the GWP used to ensure transparency [5]. In this context, when values are not available, the software provides default GWP_100_ values based on IPCC references (e.g. [6], 1995 [4], 2001 [7], 2007 [8], 2013 [9]) (Fig. 5). The GWP values will be displayed after clicking on the “execute” button. EFs (e.g. MTCO_2_E/tonne of waste managed) used in intermediary calculations of the model, are linked to the GWP reference selected by the user to ensure a consistent reporting of emissions.

### Waste generation and composition

**Fig. 6:**
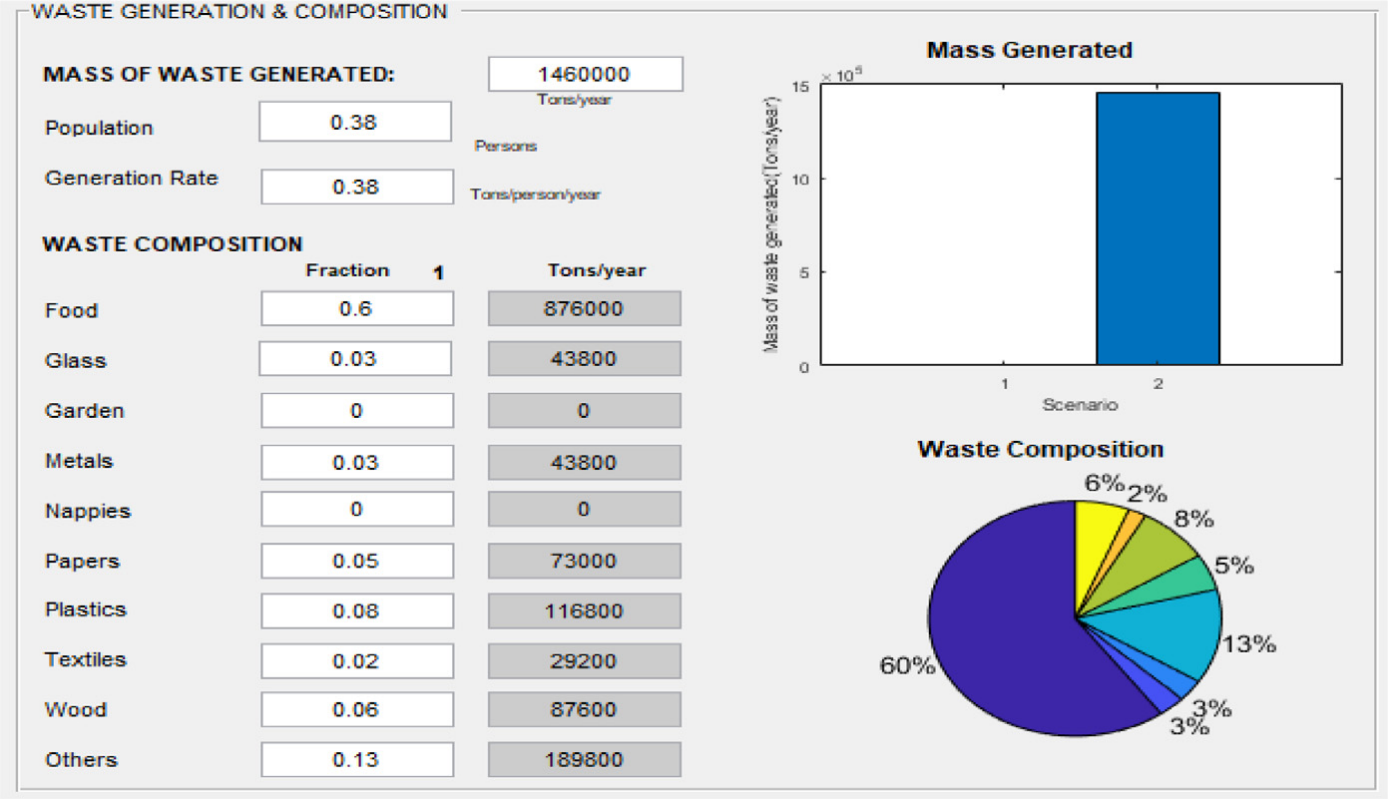
Waste generation and composition.

The user input of data related to waste generation and composition constitutes the starting point for calculating emissions and costs. The total amount of waste generated (tonnes/year) is provided by the user or extrapolated from the population (persons/year) based on per capita generation rate (tonnes/person/year) for a general study area and inventory year (Fig. 6). The user also enters the waste composition (food, glass, garden, metals, nappies, papers, plastics, textiles, wood, and others) for estimating emissions. The corresponding values are graphically displayed (Fig. 6) after clicking on the “execute” button of the “waste flow” window (“3” in Fig. 1).

## Emission accounting tool

**Fig. 7:**
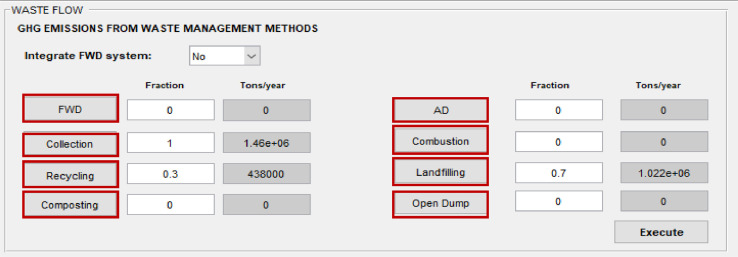
Emission accounting tool.

The SWW software accounts for emissions from various municipal solid waste (MSW) management processes including collection, sorting/recycling, biological treatment (e.g. composting and anaerobic digestion), incineration (with and without energy recovery), landfilling (with and without landfill gas collection for flaring or energy recovery), open dumping or burning. It also considers emissions from introducing a food waste disposer (FWD) system for grinding food waste at household level (Fig. 7).

Depending on the scenario definition the user has two options to account for emissions: (1) in case of selecting a “multiple case scenario” option, the software directs the user automatically to the “Optimization tool” and the “Emission accounting tool” window will be disabled in grey; (2) when the user selects a “single case scenario” option, the software offers an emission accounting tool to calculate emissions from individual waste management processes. The user first defines in the main window the amount (tonnes/year) or fraction of MSW managed under each process (Fig. 7). Values will be displayed after clicking on “Execute”.

**Fig. 8:**
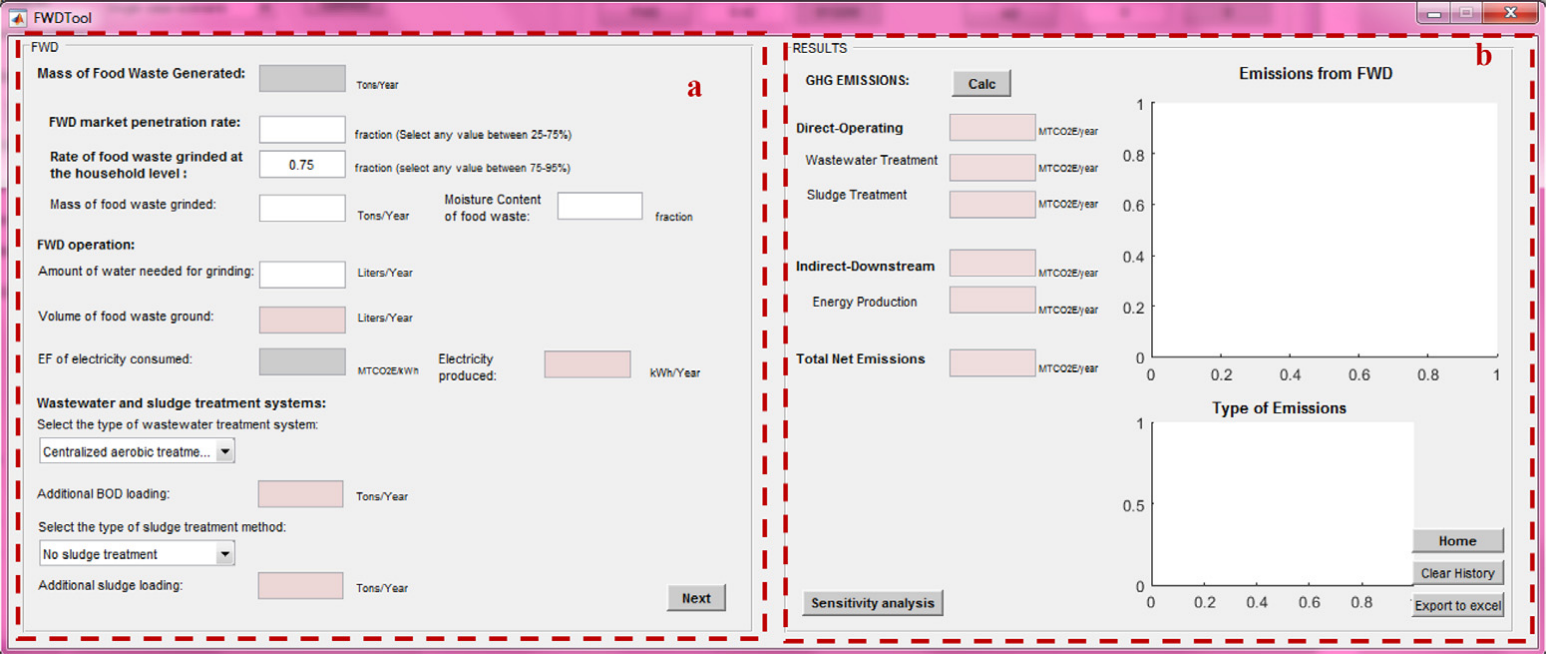
Food waste disposer (FWD) tool. *(a) Input-specific data; (b) process-specific emissions results*.

**Fig. 9:**
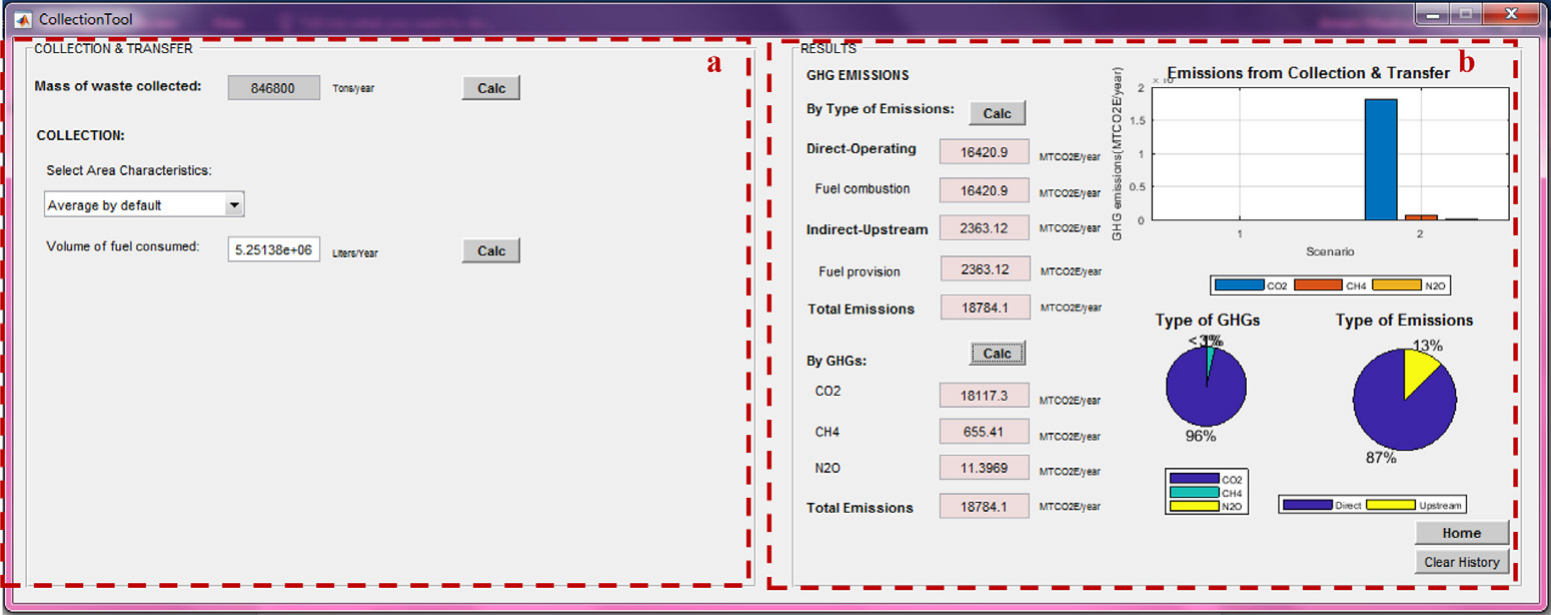
Waste collection tool. *(a) Input-specific data; (b) process-specific emissions results*.

**Fig. 10:**
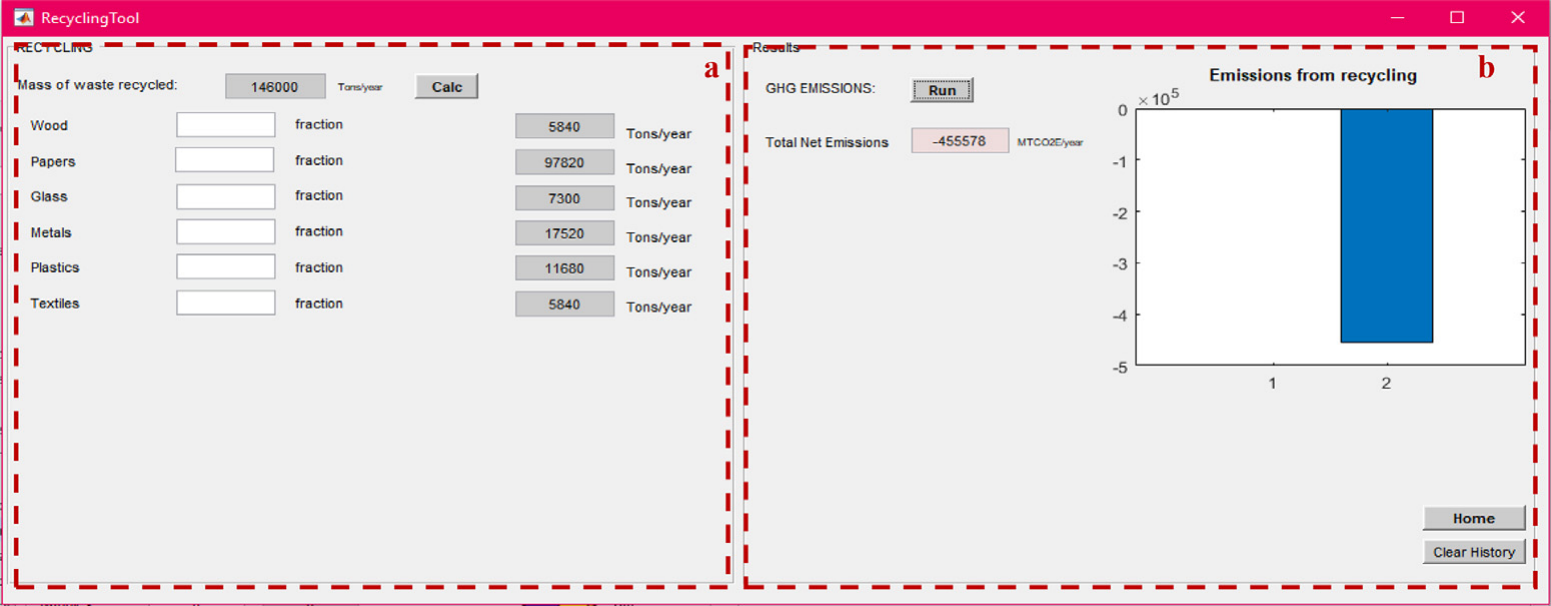
Recycling tool. (a) Input-specific data; (b) process-specific emissions results.

**Fig. 11:**
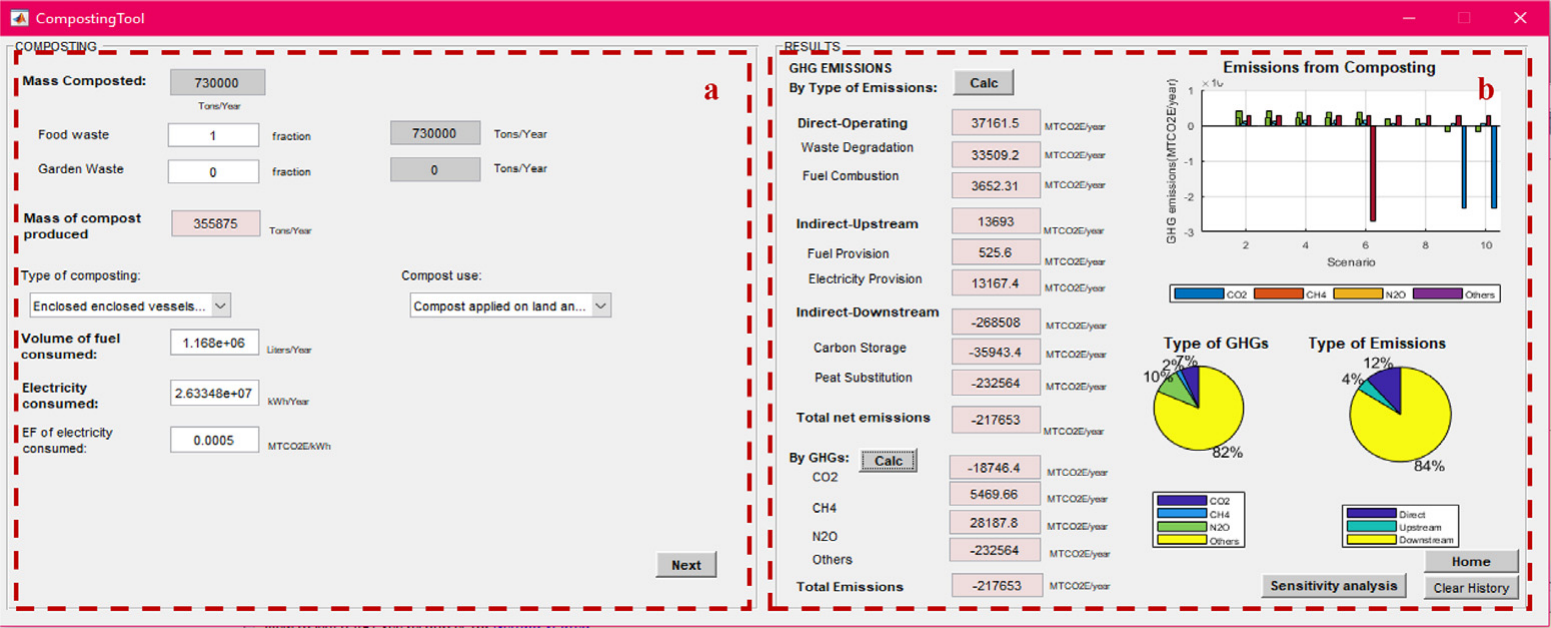
Composting tool. (a) Input-specific data; (b) process-specific emissions results.

**Fig. 12:**
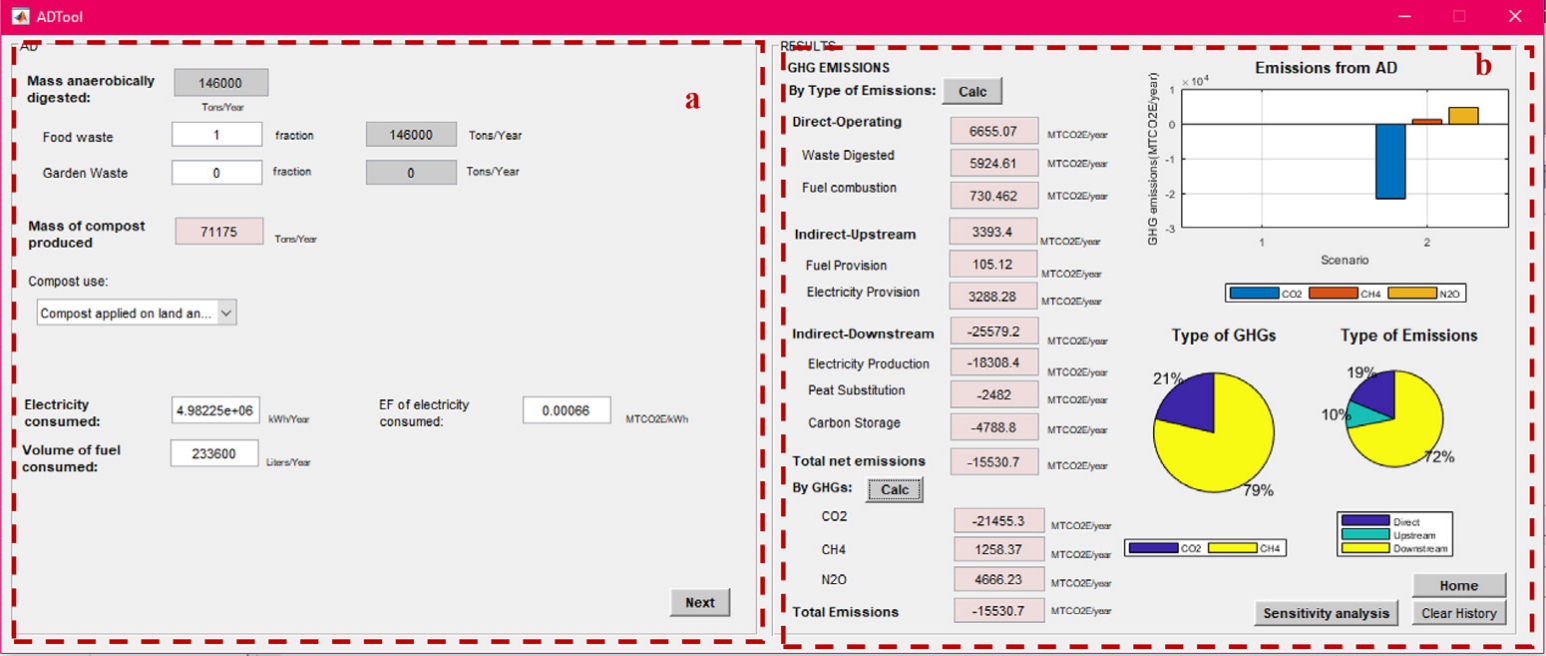
Anaerobic digestion (AD) tool. (a): Input-specific data; (b) process-specific emissions results.

**Fig. 13:**
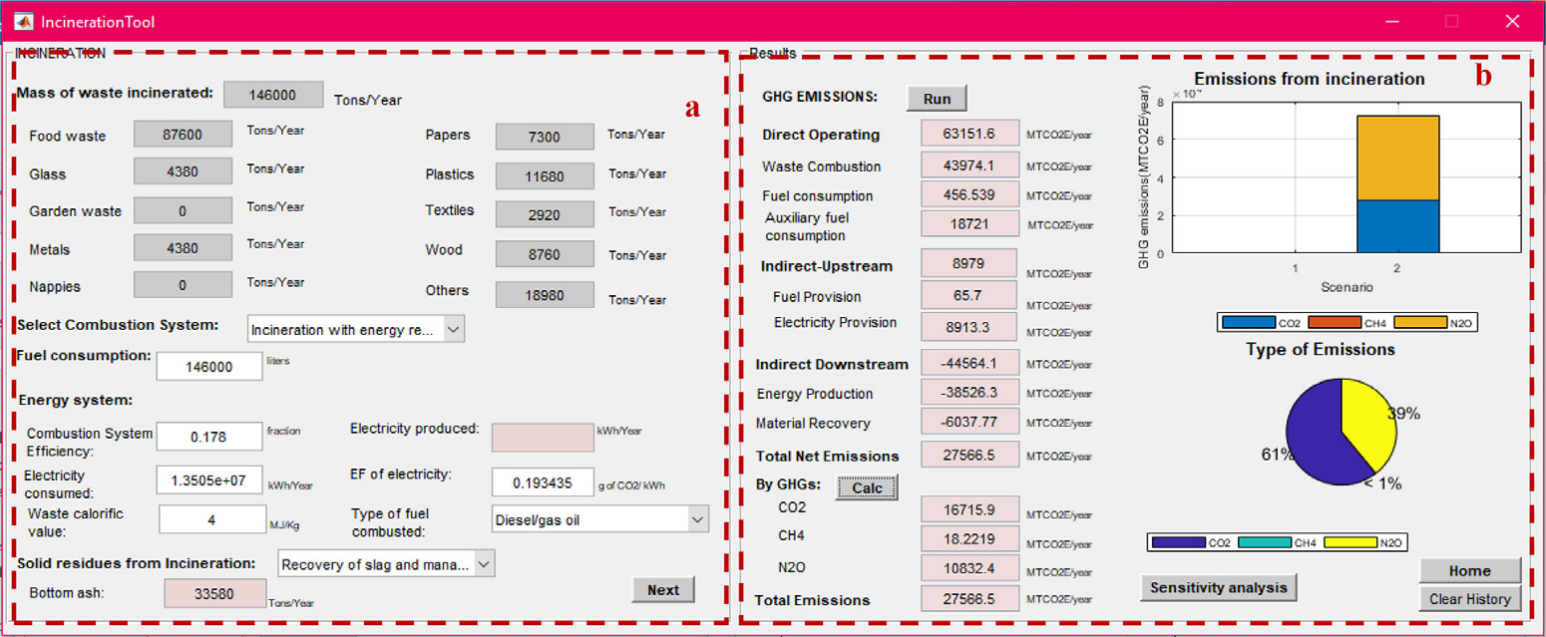
Incineration and Open burning tool. (a) Input-specific data; (b) process-specific emissions results.

**Fig. 14:**
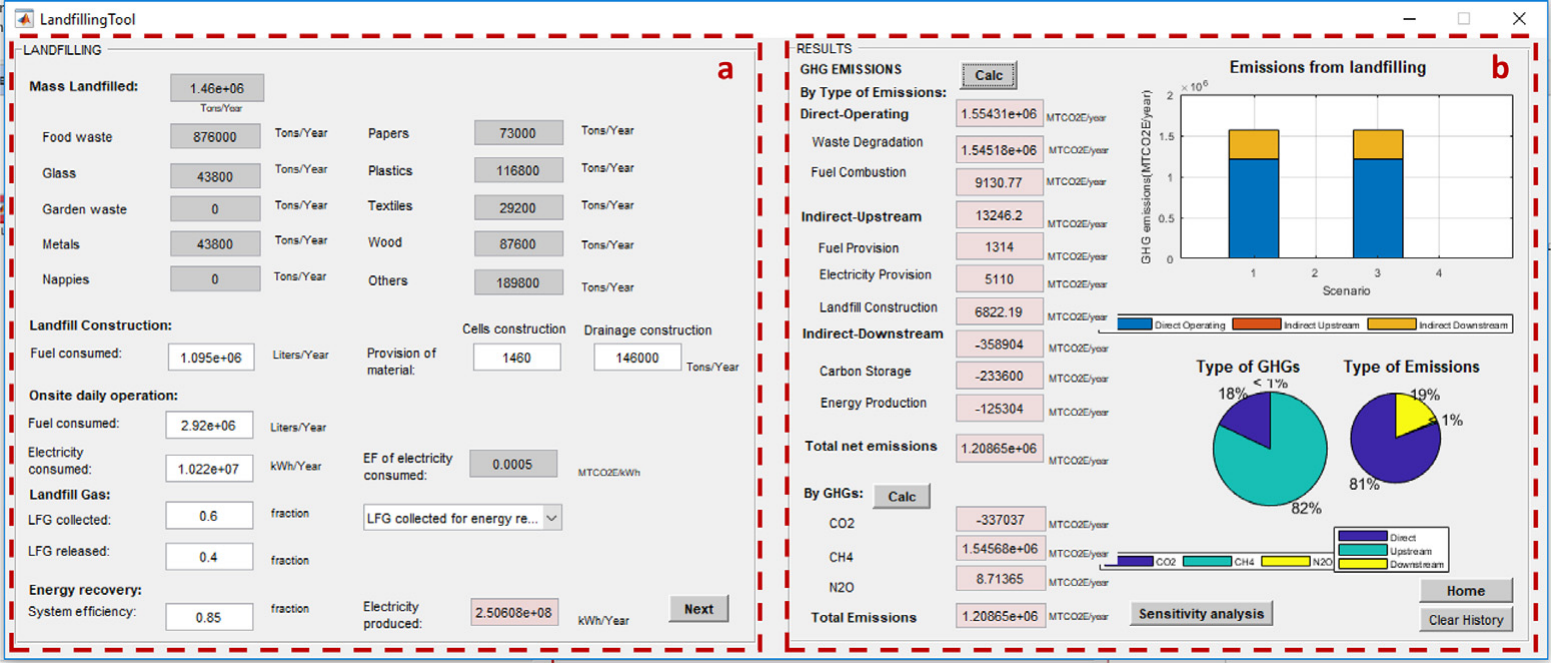
Landfilling tool. (a) Input-specific data in the landfilling process; (b) Process-specific emissions results.

**Fig. 15:**
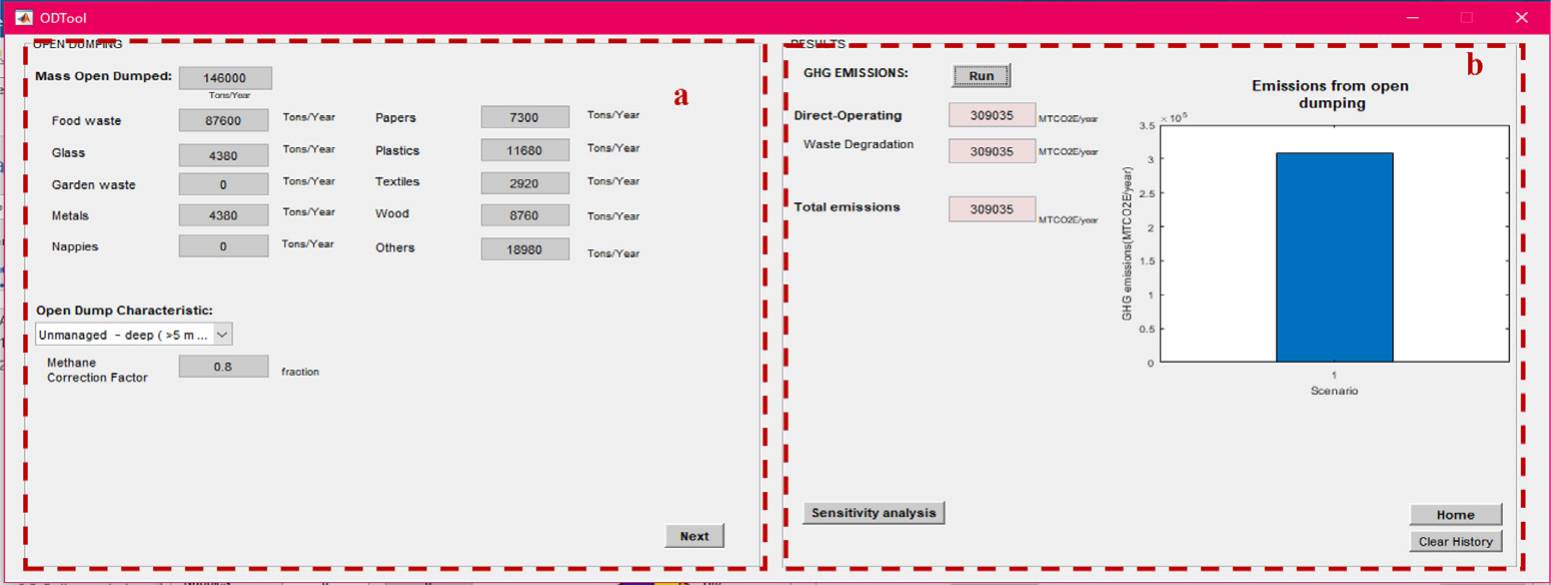
Open dumping tool. (a) Input-specific data; (b) process-specific emissions results.

After calculating the total mass of MSW managed under each method, the net total GHG emissions from individual management processes can be calculated using the “process-specific tool” template that will open by clicking on each of these processes (marked with a red box in Fig. 7). Details on the model formulation specific for each process can be found in reference [10]. Screenshots of the interface for individual process-specific tools used to calculate emissions are displayed in Fig. 8, Fig. 9, Fig. 10, Fig. 11, Fig. 12, Fig. 13, Fig. 14, Fig. 15 with detailed elaboration in the Supplementary Material.

The net total emissions estimated from waste management processes are estimated in metric tonnes of CO_2_ equivalents (MTCO_2_E) and equal to the difference between gross (Indirect-upstream and direct-operating) and avoided (Indirect-downstream) emissions.

• Indirect-upstream emissions arise from inputs of materials (e.g. provision of material for landfill construction); electricity provision (emissions occur offsite and depend on the current electricity generation mix selected by the user); and fuel provision (pre-combustion emissions associated with the extraction, processing, producing, storage, and transport of fuel).
• Direct operational emissions from system's operation are related to fuel combustion of onsite operating equipment and waste degradation as a result of physical, chemical, or biological processing (e.g. Landfill has (LFG) emissions).
• Indirect downstream emissions (or savings) are associated with avoided emissions from energy generation (depending on the selected electricity generation mix), materials substitution (e.g. recyclable materials that offsets production from virgin materials), and carbon storage.

**Fig. 16:**
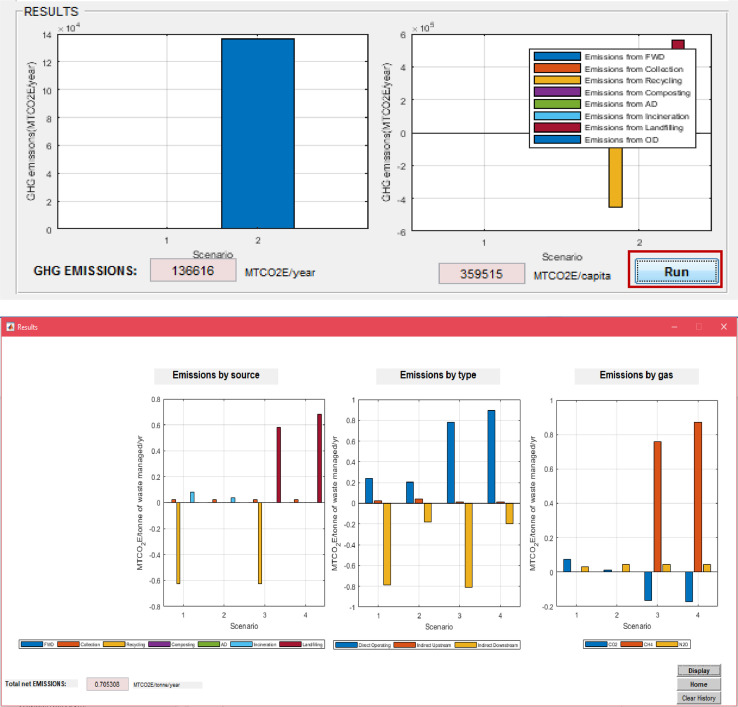
Net total emissions calculated by SWW. FWD: food waste disposer; C: collection; R: Recycling; Co: composting; AD: anaerobic digestion; I: incineration; Lf: Landfilling; OD: open dumping.

Upon finalizing the calculation of emissions specific to each process, the user must go back to the main window (Fig. 1) of SWW to display the total net emissions. The latter is displayed in total and disaggregated by type of accounting after clicking on “Run” (Fig. 16) with their corresponding graphics. A window opens to display total net emissions disaggregated by source, gas and type (Fig. 16). The software also displays the net total emissions per capita depending on the selected population number from the input data in the main window (“1” in Fig. 1).

## Economic analysis tool

**Fig. 17:**
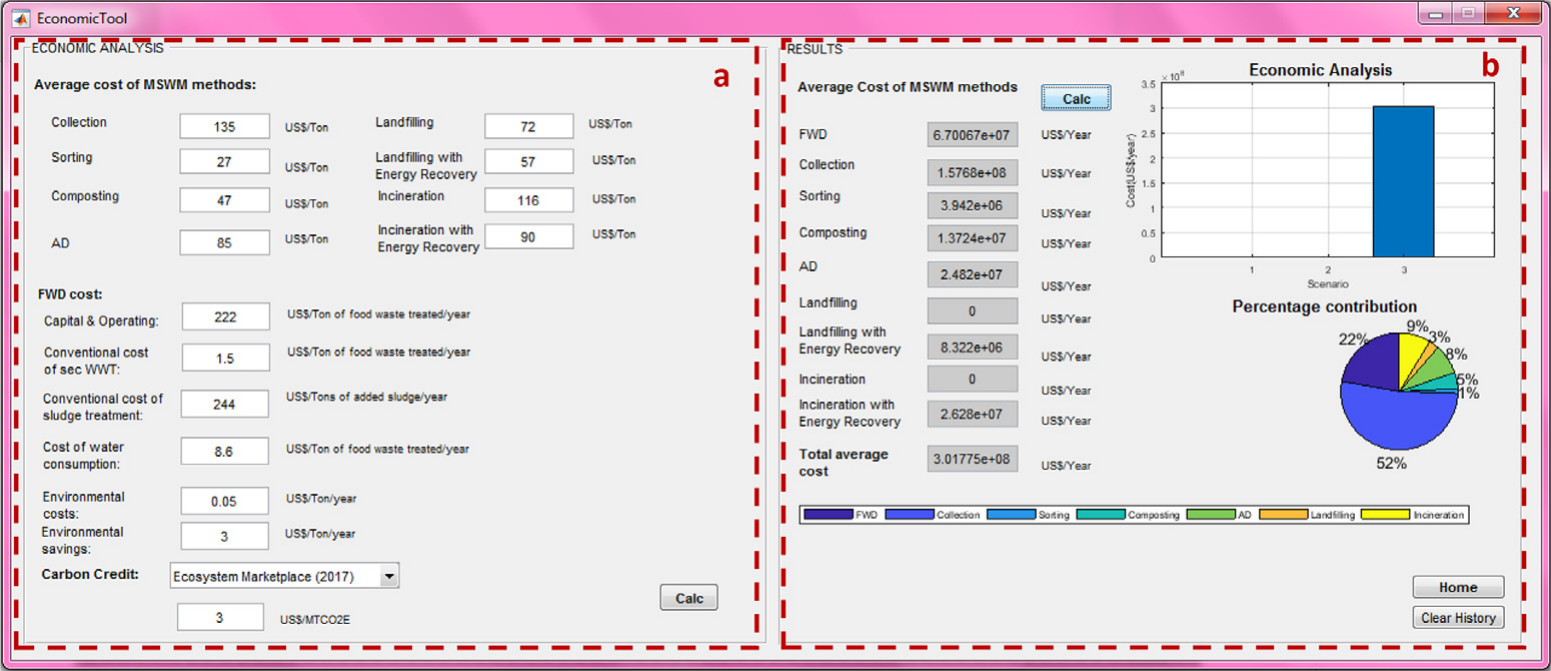
Economic analysis tool. (a) Average costs of municipal solid waste management (MSW) processes and costs of food waste disposer (FWD); (b) costs results.

When conducting a single case scenario, the user can assess the economic implications of selected waste management processes by clicking on the “Economic analysis” box (“5” in Fig. 1). Economic associations targeted the analysis of conventional (direct) and environmental (indirect) costs/savings for tested waste management systems. The conventional costs include capital and operating costs associated with management processes (“Part a” in Fig. 17). SWW provides default average operating costs (US$ per tonne of waste) of waste management processes adopted from [11,12] if data is not available (see Table 1).

**Table 1:** Average cost (US$/tonne) of MSW management processes adopted from [11,12].

	Collection	Sorting	Composting	Anaerobic digestion	Landfilling	Landfilling with energy recovery	Incineration with energy recovery	Incineration with no energy recovery
Assamoi and Lawryshyn (2012) [13]	…	…	…	…	18	…	38	…
Bianchini and Hewage (2012) [14]	…	…	…	…	56	…	…	…
Damgaard et al. (2011) [15]	…	…	…	…	70	67	…	…
Dijkgraaf and Vollebergh (2004) [16]	…	…	…	…	45	…	97	120
EC (2002) [17]	…	…	…	80	62	58	88	102
Jamasb and Nepal (2010) [18]	…	26	…	…	15	13	70	…
Kim et al. (2011) [19]	61	…	…	…	10	…	…	…
NREL (2013) [20]	…	…	…	34–90	…	…	…	…
Rabl et al. (2008) [21]	…	…	…	…	45	40	92	121
Tsilemou & Panagiotakopoulos (2006) [22]	…	…	17–73	22–67	12–50	…	117	131
Hoornweg and Bhada-Tata (2012) [23]	20–250^(^a^)^	….	5–90	20–150	10–100	…	120	…
Wrap (2016) [24]	…	28^b^	27	44	21	…	94	…
*Range (US$/Ton)*	*20–250*	*26–28*	*5–90*	*20–150*	*10–100*	*13–67*	*38–120*	*102–131*
***Average (US$/Ton)***	***135***	***27***	***47***^c^	***85***^d^	***72***^e^	***57***^e^	***90***	***116***

a Collection includes pick up, transfer, and transport to final disposal site for residential and non-residential waste.

b Cost of sorting of four waste categories or more that are delivered as comingled MSW to the material recovery facility (MRF).

c Composting excludes sale of finished compost (which ranges from 0 to 100 US$/tonne).

d Anaerobic digestion includes sale of energy from methane and excludes cost of residue sale and disposal.

e Includes an additional ~17 US$/Tonne of waste for onsite leachate and gas collection, treatment and management [14,17].

The user can enter capital costs associated with constructing new facilities that are considered as part of a new waste management decision. With the exception of landfilling whereby capital (e.g. construction) costs are amortized into their operating costs because they are considered as an ongoing construction process. The cost of MSW management is estimated by multiplying the average costs (US$ per tonne) of alternatives by the total amount of waste managed (“Part b” in Fig. 17). SWW also allows the user to visualize tested scenarios and shows the percentage contribution of each waste management process to the total cost (“Part b” in Fig. 16). The cost of introducing FWDs includes (1) capital/operating costs, (2) costs of managing additional wastewater and sludge loads, and (3) the cost of increased consumption of domestic water for grinding the food waste (“a” in Fig. 17) with electricity cost for operation of FWDs being negligible. Environmental savings comprise costs forgone due to the decrease in requirements for managing food wastes diverted from the waste stream such as leachate and gas management [11].

The offset of emissions was quantified based on the carbon market. SWW allows the user to define the average price or to select from different values reported by the Ecosystem Marketplace from 2010 to 2017 [25] from the drop-down menu (“Part a” in Fig. 17). The average value is used to assess associated benefits and allows the estimation of minimal savings when the carbon footprint is reduced through regulated and voluntary global markets for offsetting of carbon credits.

Following that, the user may go back to the main window, total cost including and excluding carbon credits will be displayed after clicking on the “Execute” button (“5” in Fig. 1).

## Optimization tool

**Fig. 18:**
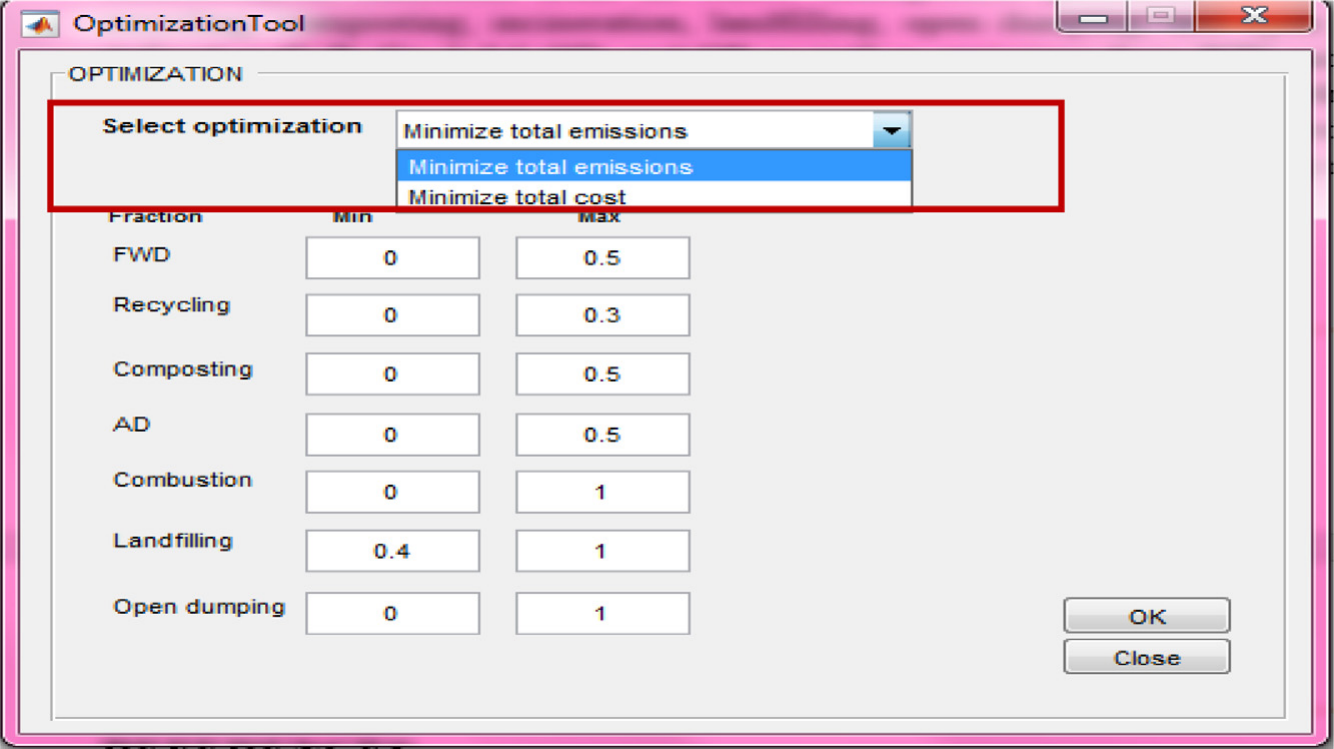
Optimization tool.

SWW offers an optimization tool based on linear programming (LP) to provide decision-makers with optimum integrated waste management systems for any region. The emissions structure allows the software to optimize following a life cycle inventory approach, while considering economic implications including carbon credit and corresponding costs of future management systems and policies. Accordingly, the user has the option to conduct the optimization based on minimal total emissions or costs. This can be selected from the drop-down menu marked with a red box in Fig. 18.

User-specified constraints can be introduced by setting the minimum and maximum fractions of waste under a specific management process to examine specific policies or set specific targets (Fig. 18). After completing all input data in the optimization tool, the user clicks on “Ok” (Fig. 18). In addition, the user must introduce other input data such as the scope of accounting, GWP, mass of waste generated, and waste composition (“1” in Fig. 1). The waste flow window (“3” in Fig. 1) is disabled. Then the user clicks on “Run” from the main window (“4” in Fig. 1). The resulting optimal waste management system with corresponding fractions of MSW under waste management processes will be displayed in as “4” in Fig. 1.

Note that this tool is launched once the user selects the “multiple case scenario” from (“1” in Fig. 1). The user can always click on “Optimize” (“2” in Fig. 1) to change constraints’ values or the optimization method and then click on “Run” to run the model again. SWW allows a graphical visualization of simulation results (“4” in Fig. 1) when running the optimization tool. In case of running an optimization based on minimizing total emissions, the user must click again on the “economic analysis” tool that will calculate total costs based on the optimized waste management system and will also display the total net with/without carbon credit (“5” in Fig. 1) after clicking on “Execute”. On the other hand, if the user selects the optimization based on minimal costs, the resulting emissions and costs including carbon credits will be displayed directly as in “4” and 5” in Fig. 1.

## Sensitivity analysis tool

**Fig. 19:**
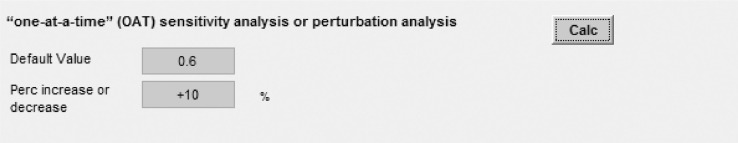
One at a time sensitivity analysis.

**Fig. 20:**
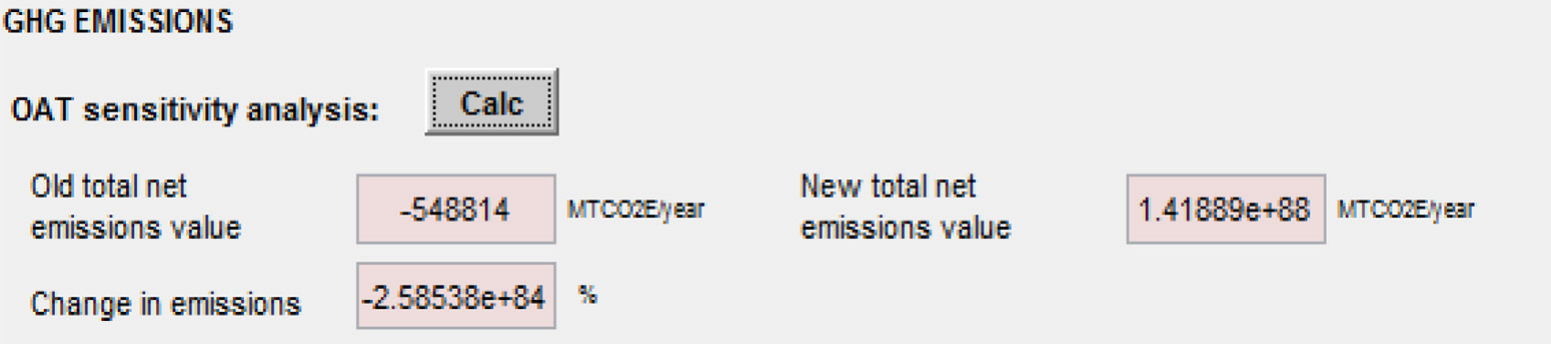
Results of an OAT analysis.

**Fig. 21:**
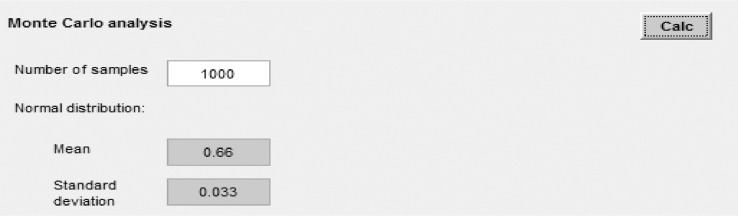
Monte Carlo analysis. Parameters are simulated as a normal distribution around their means with a standard deviation of 5% (or 95% confidence interval at +/−10%).

**Fig. 22:**
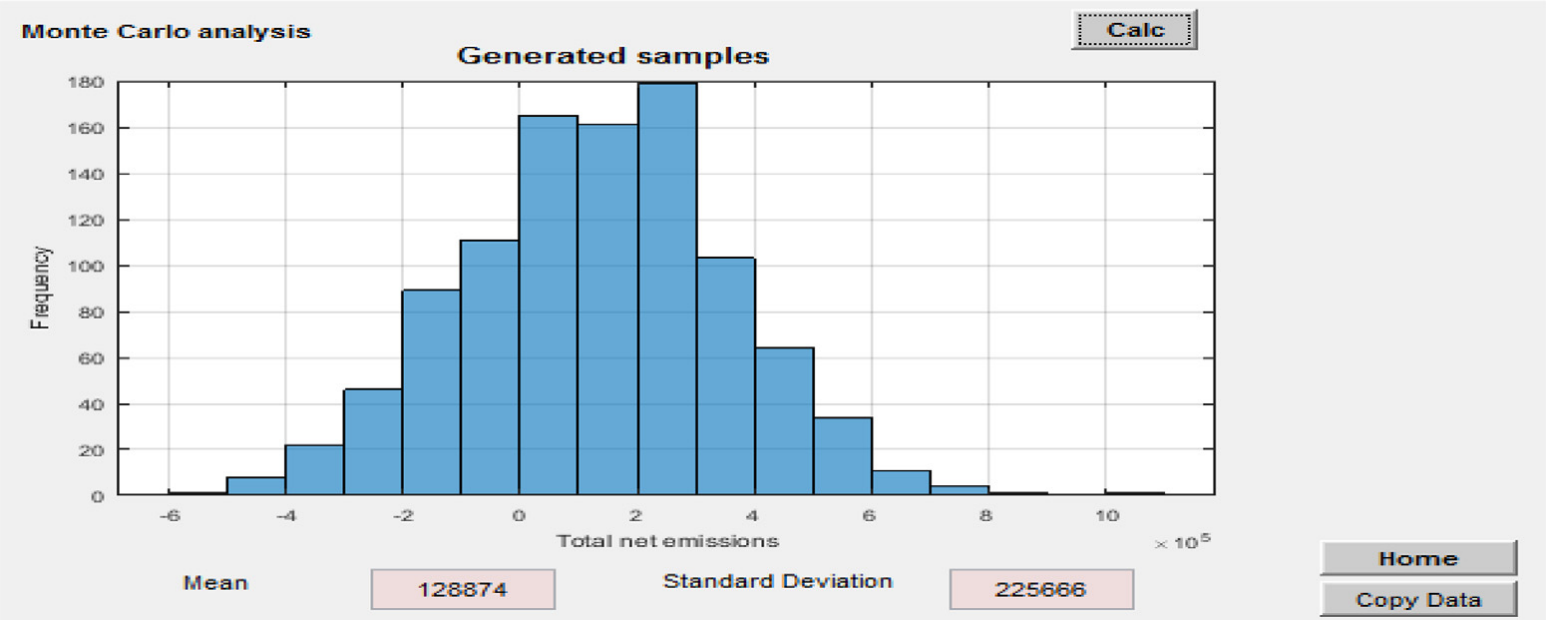
Monte Carlo simulation results.

**Fig. 23:**
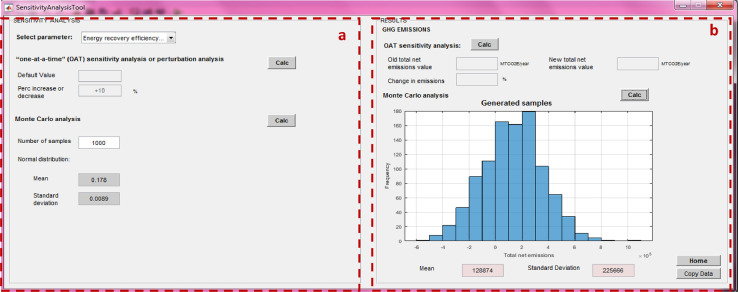
Sensitivity analysis tool. *(a) Input data, (b) display of results*.

SWW allows the user to select key parameters for sensitivity and uncertainty analysis through the use of a “Sensitivity analysis” tool (Fig. 23) whereby each parameter can be individually modified to assess its impact on emissions by following two methods:

(1) The One-at-a-time (OAT) analysis: the user specifies the percent increase or decrease in the initial value of a parameter with the results displayed as percent change in emissions. The OAT assesses the influence of each parameter based on the same initial variation (Fig. 19) with the corresponding results displayed after clicking on “Calc” (Fig. 20).

(2) Monte Carlo analysis: to calculate the uncertainty of the obtained results, the sensitivity analysis tool considers representation of parameter uncertainties as probability distributions and propagation by Monte Carlo simulation [26]. The user can define, for each parameter, a probability distribution of normal shape. For a Monte Carlo simulation, the calculation involves sampling the normal distribution to obtain a list of values for each parameter (the length of which equals the number of runs) and then running the model with this list of values. To obtain a first rough impression of the results of the Monte Carlo simulation, results are first run with a list of a sampled values (e.g. 1000 runs) for the normal distribution (Fig. 21). The result of this first run is thus imprecise but quick to calculate, which allows the user to gain immediate feedback on the effect of using the distribution. Instead of showing the list of sampled values in the result, which can be very long, the results displayed graphically (as a histogram as depicted in Fig. 22 after clicking on the “Calc” button with the mean and standard deviation of the list of values in the results fields. In addition, the user may want to obtain more precise results, e.g. for certain impact categories' impacts, and run the simulation with a larger list size, e.g. 10,000 runs. This can be done by clicking on “Number of samples” and chose for example “10,000 runs”, which will then run the Monte Carlo simulation 10,000 times. The user may export the corresponding list of resulting values by clicking on “copy data”, which can then be pasted into Excel for further analysis. The sensitivity analysis using the Monte Carlo simulation in this study was based on the recommended method by [26].

## Policy analysis tool

**Fig. 24:**
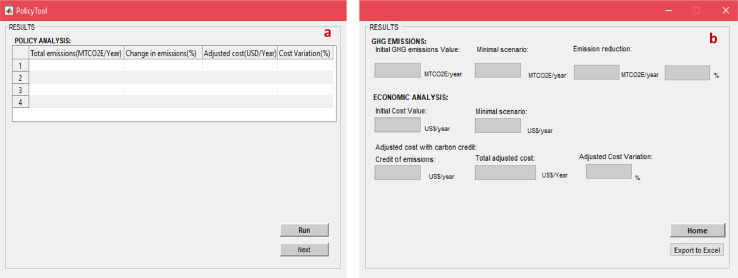
Policy analysis tool. *(a) Displayed input data; (b) results*.

SWW addresses multi-objectivity by considering environmental valuation in the form of carbon externalities offering a “Policy analysis tool” (Fig. 24). The carbon credit expressed in US$ per MTCO_2_E are assigned to environmental emissions. The ultimate objective is to evaluate scenarios based on minimizing total net emissions or costs while considering implications in terms of carbon credit for both cases. This can be of interest at the policy planning level by influencing emissions reporting targets under the United Nations framework convention on climate change (UNFCCC) commitments or affect reduction targets/ mitigation measures using carbon credits to meet nationally determined contributions (NDCs) under the Paris Agreement for example.

SWW keeps track of evaluated scenarios under the “single case scenario” options (Fig. 24(a)). The result of the baseline scenario (first evaluated scenario) is used to test the impact of policy options on emissions. It also depicts cost variations achieved under each scenario as percentages of existing costs under the baseline scenarios based on average conventional and environmental costs including carbon credits. The results are displayed in Fig. 24(b) after clicking on “Run” and then “Next” buttons. The results can also be exported into an excel file by clicking on “Export to excel” from the main window.

## Declaration of Competing Interest

The authors declare that they have no known competing financial interests or personal relationships that could have appeared to influence the work reported in this paper.

## Appendix Supplementary materials

Image, application 1
