# Supplementary material for: Optimizing emissions and carbon credit from integrated solid waste and wastewater management: A MATLAB-based model with a Graphical User Interface (v1)
Source: MethodsX. 2020 Feb 24;7:100839. doi: 10.1016/j.mex.2020.100839 (PMC7078370; doi:10.1016/j.mex.2020.100839)
Supplement: Supplementary file 1 [file mmc1.docx]

**Optimizing Emissions and Carbon Credit from**

**Integrated Solid Waste and Wastewater Management:**

**A MATLAB-based model with a Graphical User Interface (v1)**

Amani Maalouf*, Mutasem El-Fadel
Department of Civil & Environmental Engineering, American University of Beirut, Lebanon

* Corresponding author: [ahm22@mail.aub.edu](mailto:ahm22@mail.aub.edu)

# Supplementary Material

# Emission accounting from individual waste management processes

The Solid waste and wastewater-SWW management software offers individual process-specific tools used to calculate emissions as detailed below. The software keeps track of mass and material flows specific for each process. The mass of waste treated is based on the fraction of MSW that is diverted to a specific process as defined by the user (“3” in Figure M1). The user must then input the amount of fuel and electricity consumed specific to each process. In case these values are left empty, SWW provides default values. The total emissions are disaggregated by type of emissions by clicking on “Calc”. The user can always clean the graphs history by clicking on the “Clear History” button and go back to the software main window by clicking on “Home”. The following subsections describes the template processes available in the software.

## FWD

The user must first select from the drop-down menu of the main window (in Figure SM 1) whether to introduce “yes” or not “no” the food waste disposer (FWD) system at the household level. This is very important in order to deduct the mass of the food waste grinded and diverted from the solid waste collection system to the wastewater (WW) collection system. Then the user must select the desired fraction of municipal solid waste (MSW) to be diverted to the WW management system. The FWD-specific tool will open after clicking on the “FWD” button in the main emission-accounting tool window (Figure SM 1).


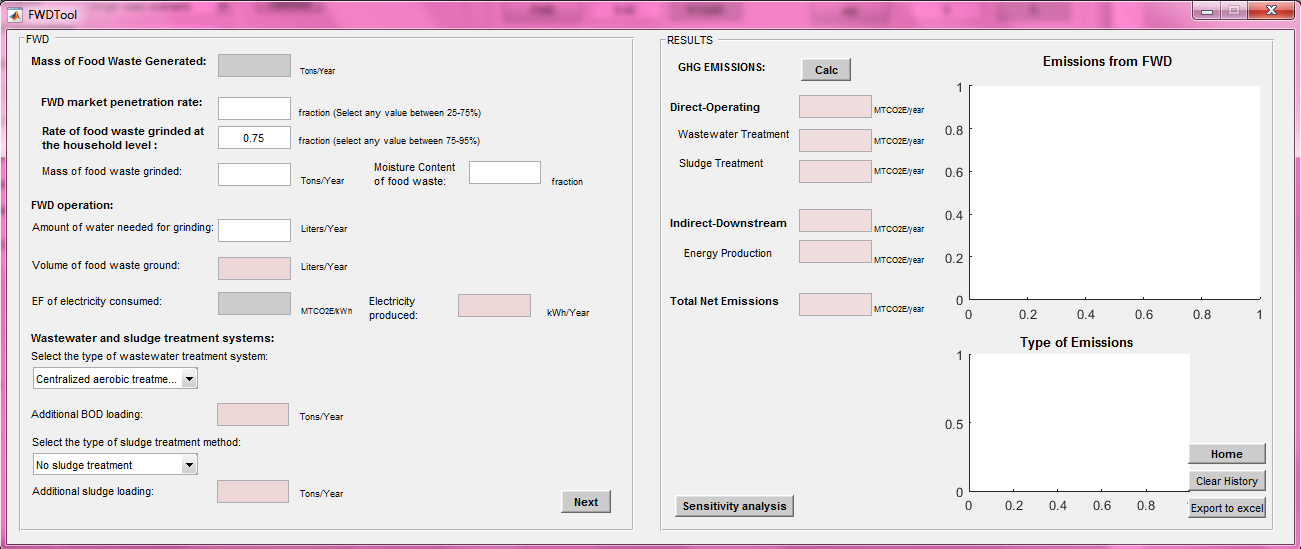


**a**

**b**

Figure SM 1. Food waste disposer (FWD) tool
a: Input-specific data; b: Process-specific emissions results

Part “a” in Figure SM 1 considers all input-specific data for the FWD policy:

1. The software keeps track of the mass and material flows specific to each process as displayed in Figure SM 2.


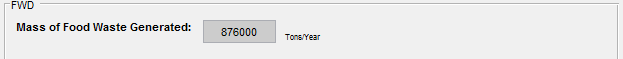


Figure SM 2. Mass of waste generated

1. The fraction of the waste diverted to the WW system through the use of FWD is dependent on (Figure SM 3):

- The FWD market penetration that can typically vary between 25 to 75%
  (based on literature reported values/guidelines and past experience [1]),
- The amount of food waste grinded at the household level that can typically range between 75 to 95%
  (based on literature reported values/guidelines and past experience [1]).


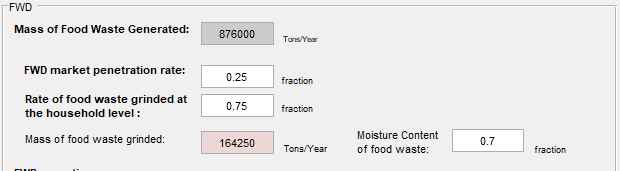


Figure SM 3. FWD market penetration rate and amount of waste grinded

Note that these ranges are based on literature reported values/guidelines and past experience [13], yet, the software is not constrained by these ranges and the user can select any value according to the targeted policy. If the market penetration rate (*m*) value is left empty, the SWW considers the maximum rate of food waste ground (z=0.95) and calculates *m* based on the selected FWD fraction such as:

m= (Total mass of waste generated x Fraction of MSW that is diverted through the use of FWD)/ (Rate of food waste ground x Total mass of food waste generated).

The software then calculates the mass of waste grinded and displays the value in the pink box (marked in red in Figure SM 3).

1. The software also requires the input of the food waste’ moisture content. If the latter is left empty, SWW displays a default value of 0.7 (Figure SM 3).
2. The user also has to input specific data related to the operation of the FWD such as the “Amount of water needed for grinding”; the “Volume of food waste ground”; the “Emission factor of electricity consumed” and “Amount of electricity produced”. In case these values are left empty, SWW calculates and displays theses values by default (Figure SM 4).


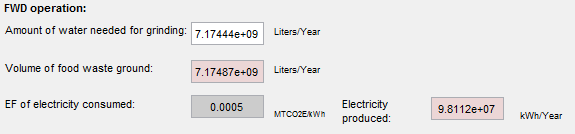


Figure SM 4. Data input for the FWD operation

1. The WW management system may consist of aerobic (e.g. centralized aerobic treatment plant) or anaerobic processes (e.g. anaerobic lagoon, septic system) for treating the additional biochemical oxygen demand (BOD) load that is calculated by default and displayed in the pink box (marked in red in Figure SM 5) such as:

*BOD = (Mass of food waste generated * Rate of food waste ground * market penetration rate) (Average Moisture content + Volume of water needed to grind 1 Ton of organic food) * Average concentration of BOD of food waste based on experimental results * Water density*

The WW system may be selected from the drop-down menu as shown in Figure SM 5.

| 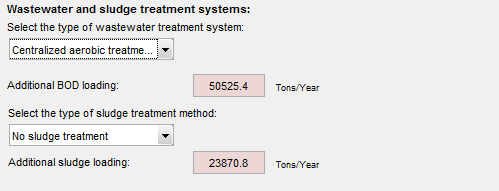 | 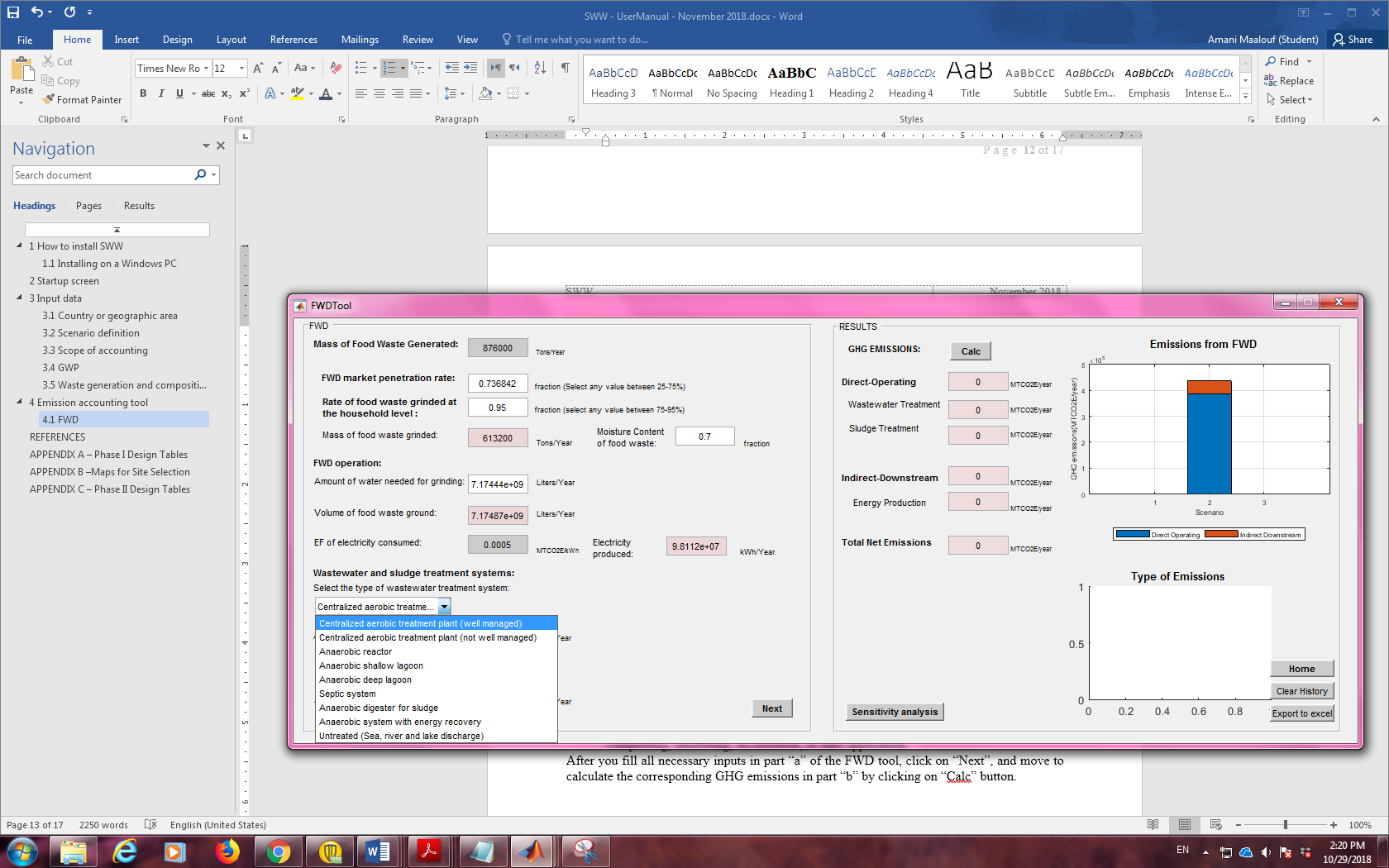 |
| --- | --- |

Figure SM 5.Select the wastewater treatment system

1. The software offers several sludge management (SM) options including anaerobic digestion, composting, landfilling, incineration, or land application as shown in the drop-down list in Figure SM 6. The additional sludge (S) is also calculated by default with corresponding value displayed in the pink box (marked in red in Figure SM 6) such as:

*S= (Mass of food waste generated * Rate of food waste ground * market penetration rate) (Average Moisture content + Volume of water needed to grind 1 Ton of organic food) * Average concentration of settable solids of the food waste based on experimental results * Water density*

| 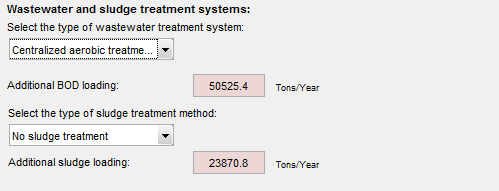 | 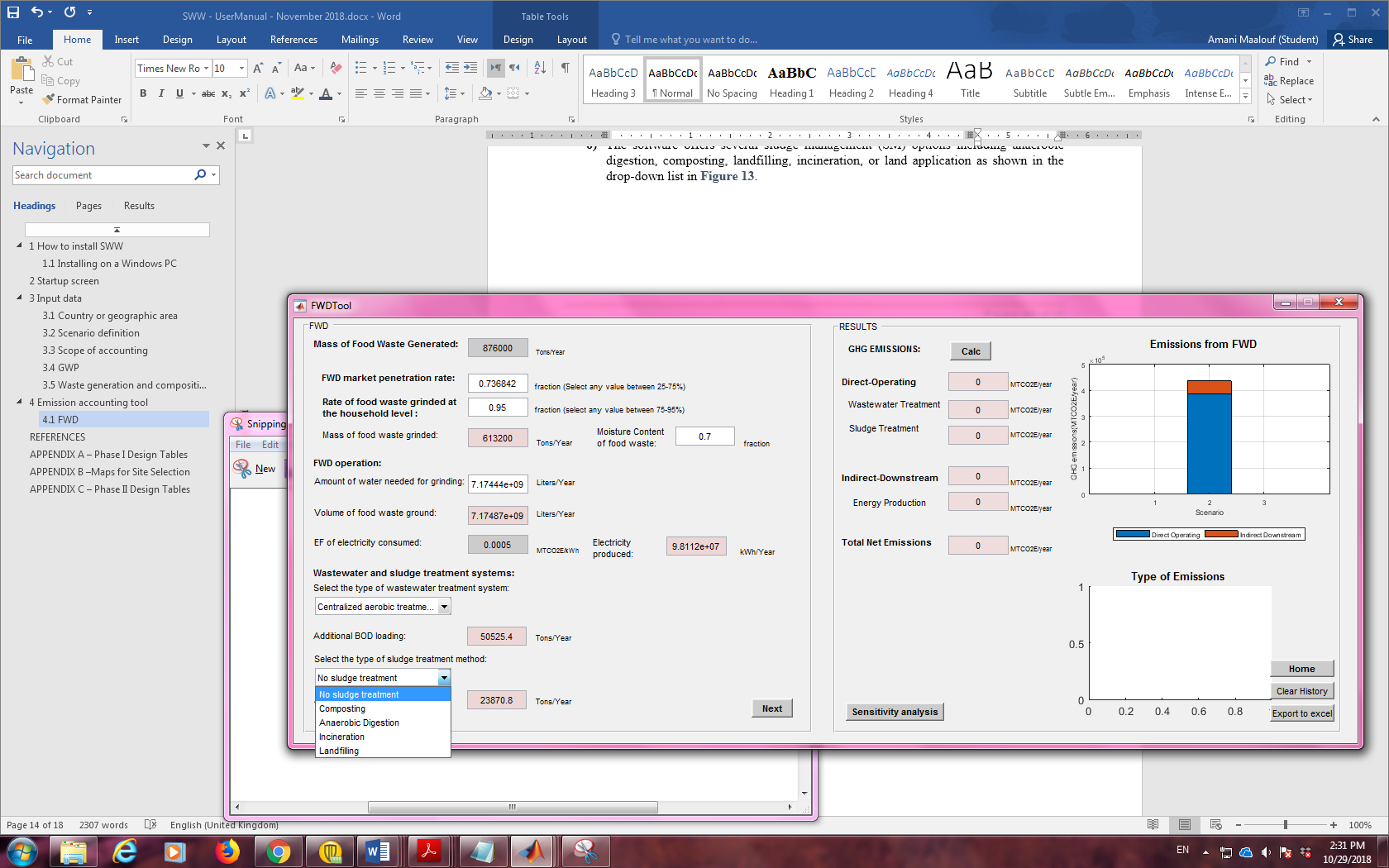 |
| --- | --- |

Figure SM 6. Select the sludge management option

After filling required inputs in part “a” of the FWD tool, the user clicks on “Next”, and proceeds to calculate the corresponding GHG emissions in part “b” by clicking on the “Calc” button (Figure SM 1). The total net emissions from introducing a FWD are disaggregated by type of emissions: 1) Direct-operating emissions associated with wastewater and sludge treatment; and 2) Indirect-downstream emissions or savings from energy production. The results are also displayed graphically to allow the user to visualize the independent percentage contribution from direct and indirect processes (Figure SM 7). The history of displayed figures can be cleared by clicking on the “Clear History” button. The user may also export the results into an excel file by clicking on “Export to excel”. Finally, by clicking on the “Home” button the user can go back to the startup window of the software to calculate emissions from other waste management processes.


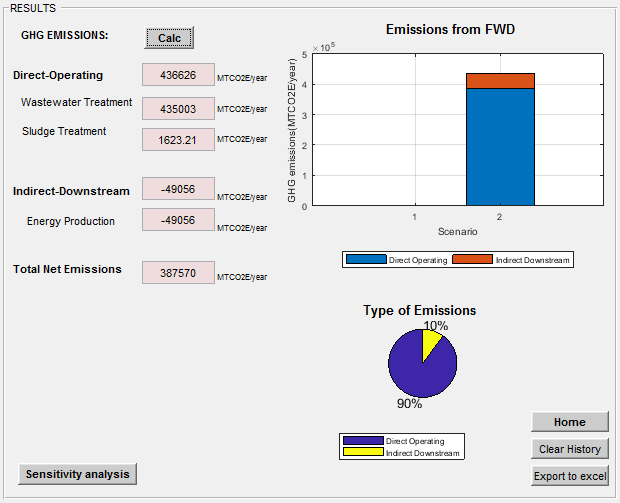


Figure SM 7. Emissions results of FWD system

## Waste collection

The waste collection tool is presented in Figure SM 8. The software keeps track of the mass and material flows specific to each process as displayed in Figure M16 whereby the mass of the waste collected can be calculated by clicking on the “Calc” button. The latter is calculated based on the waste collection rate selected by the user in the main window (“3” in Figure M1).


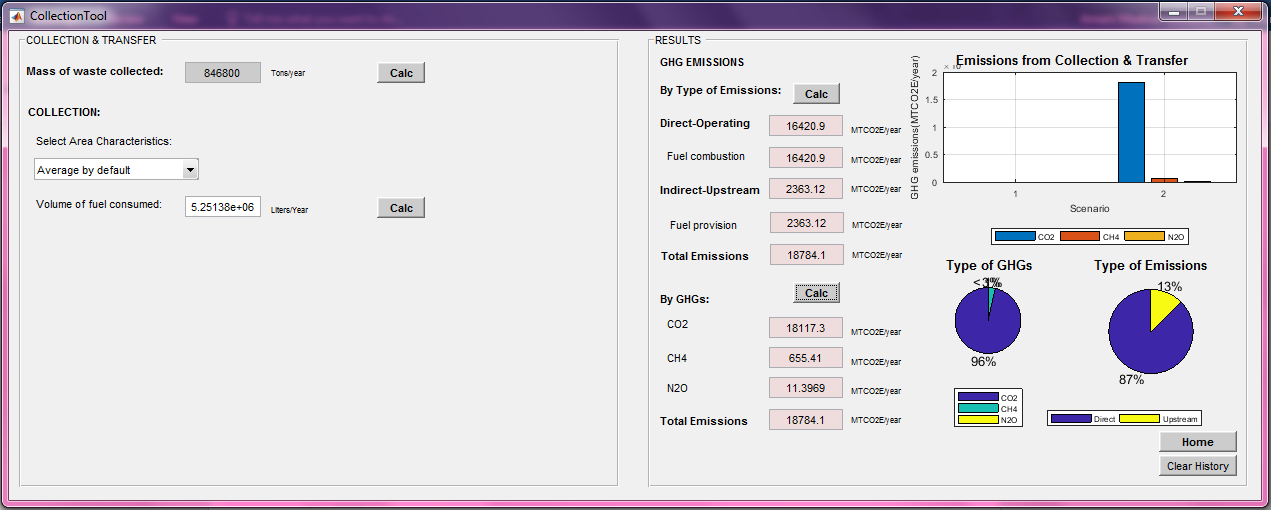


**a**

**b**

Figure SM 8. Waste collection tool
a: Input-specific data; b: Process-specific emissions results


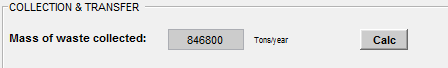


**Figure SM 9.** Mass of waste collected

Depending on the tested area characteristics, which can be selected by the user from the drop-down list in Figure SM 10, SWW calculates the volume of fuel consumed during the collection process. The user also has the option to directly insert the total volume of fuel consumed irrespective of the area characteristic or to select the default average, which is based on literature reported values and then click on “Calc”.


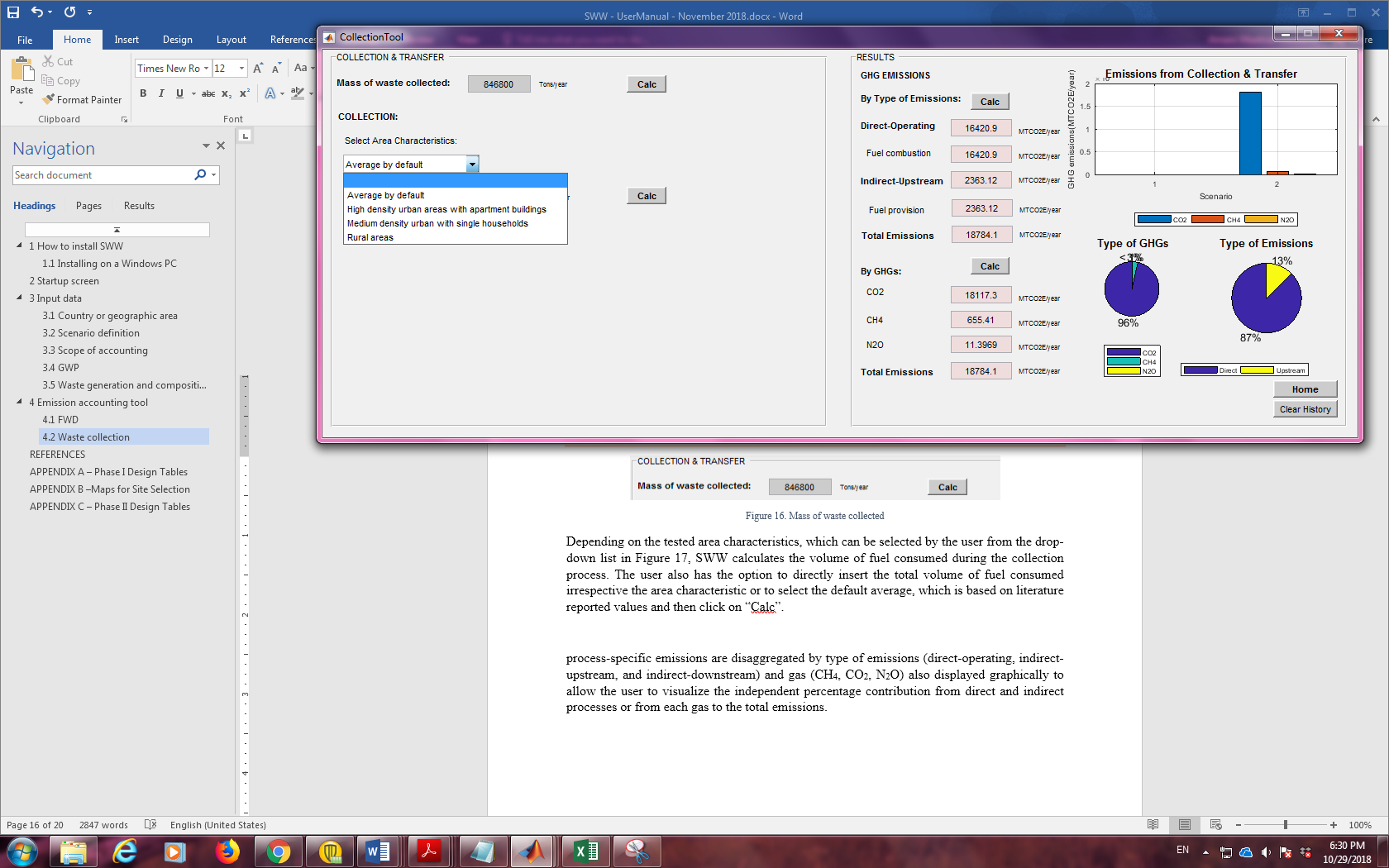


Figure SM 10. Select test area characteristic

The total emissions are disaggregated by type of emissions by clicking on “Calc” (Figure SM 11): 1) direct-operating from fuel combustion of waste collection vehicles during collection process; and 2) indirect-upstream from fuel provision. The results are also displayed by gas (CH_4_, CO_2_, N_2_O) as shown in Figure SM 12. The user can always clean the graphs history by clicking on the “Clear History” button and go back to the software main window by clicking on “Home”.


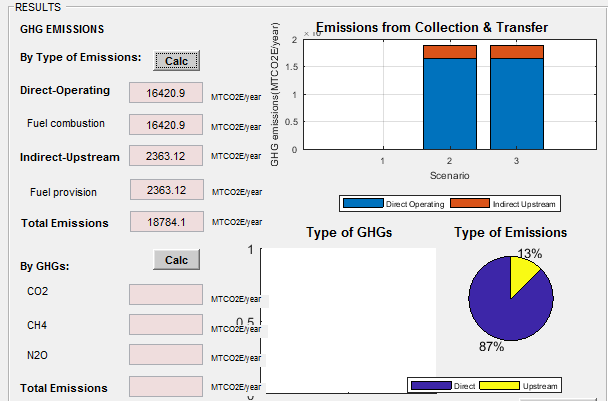


Figure SM 11. Emissions from collection disaggregated by type


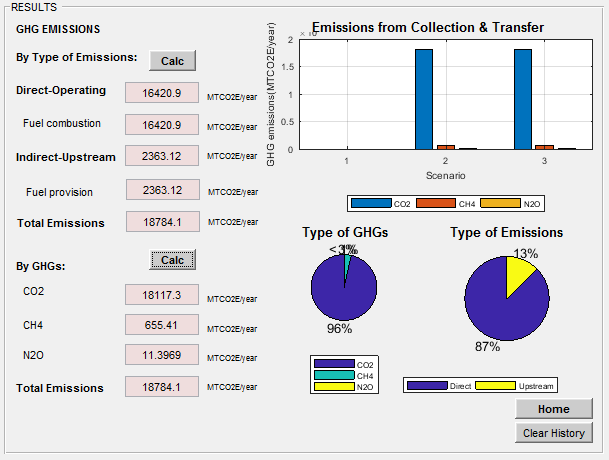


Figure SM 12.Emissions from collection disaggregated by gas

## Recycling

The software keeps track of the mass and material flows specific to each process as displayed in Figure SM 13. The mass of waste recycled is based on the fraction of MSW that is diverted to recycling as selected by the user in the main window (“3” in Figure M1). The user must input the fractions of waste materials recycled (as marked with red box in Figure SM 13). In case the fractions are left empty, SWW uses values adopted from [2] to calculate the mass of recycled materials. All masses are calculated and displayed in grey boxes by clicking on the “Calc” button.


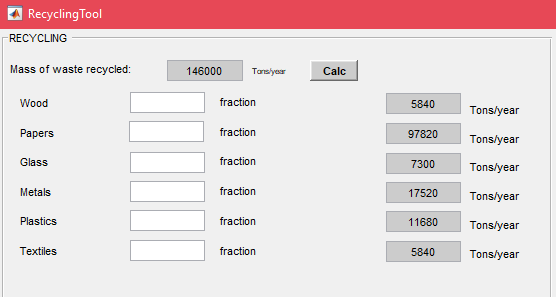


Figure SM 13. Input data of the recycling process

Direct emissions from remanufacturing of recyclables and indirect (avoided) emissions from virgin material manufacturing are combined to estimate recycling net total emissions or savings as shown in Figure SM 14. Note that SWW assumes a closed-loop recycling process whereby the end-of-life product is recycled into the same product.


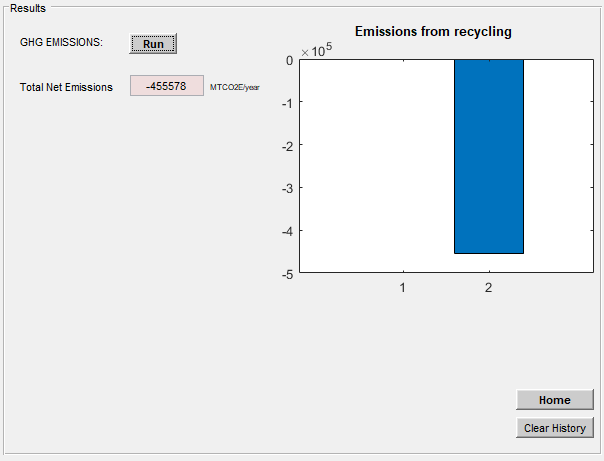


Figure SM 14. Net total emissions from recycling

## Composting

The software keeps track of the mass and material flows specific to each process as displayed in Figure SM 15. The mass of waste composted is based on the fraction of MSW that is diverted to composting as selected by the user in the main window (“3” in Figure M1). The user must input the fraction of waste materials composted (marked with a red box in Figure SM 15). In case the fractions of waste composted are left empty, by default, SWW considers that food waste constitutes 100% of waste composted. SWW will then automatically calculates the total mass composted, waste materials composted, and mass of compost produced Figure SM 15.


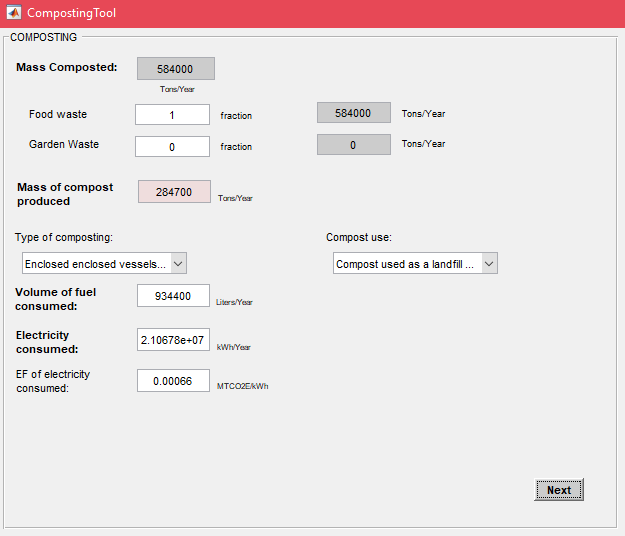


Figure SM 15. Mass input data for composting process

The user must then input the amount of fuel and electricity consumed for the operation of the composting process. In case these values are left empty, SWW calculates them based on the selected composting technology from the drop-down menu marked in the red box in Figure SM 16. Select composting technology. After filling the required input data, the user clicks on the “Next” button to display all values and proceeds to calculate net total emissions.

| 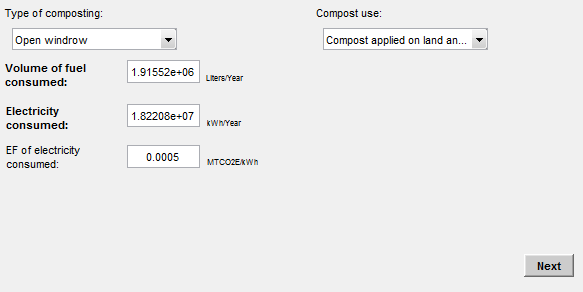 | 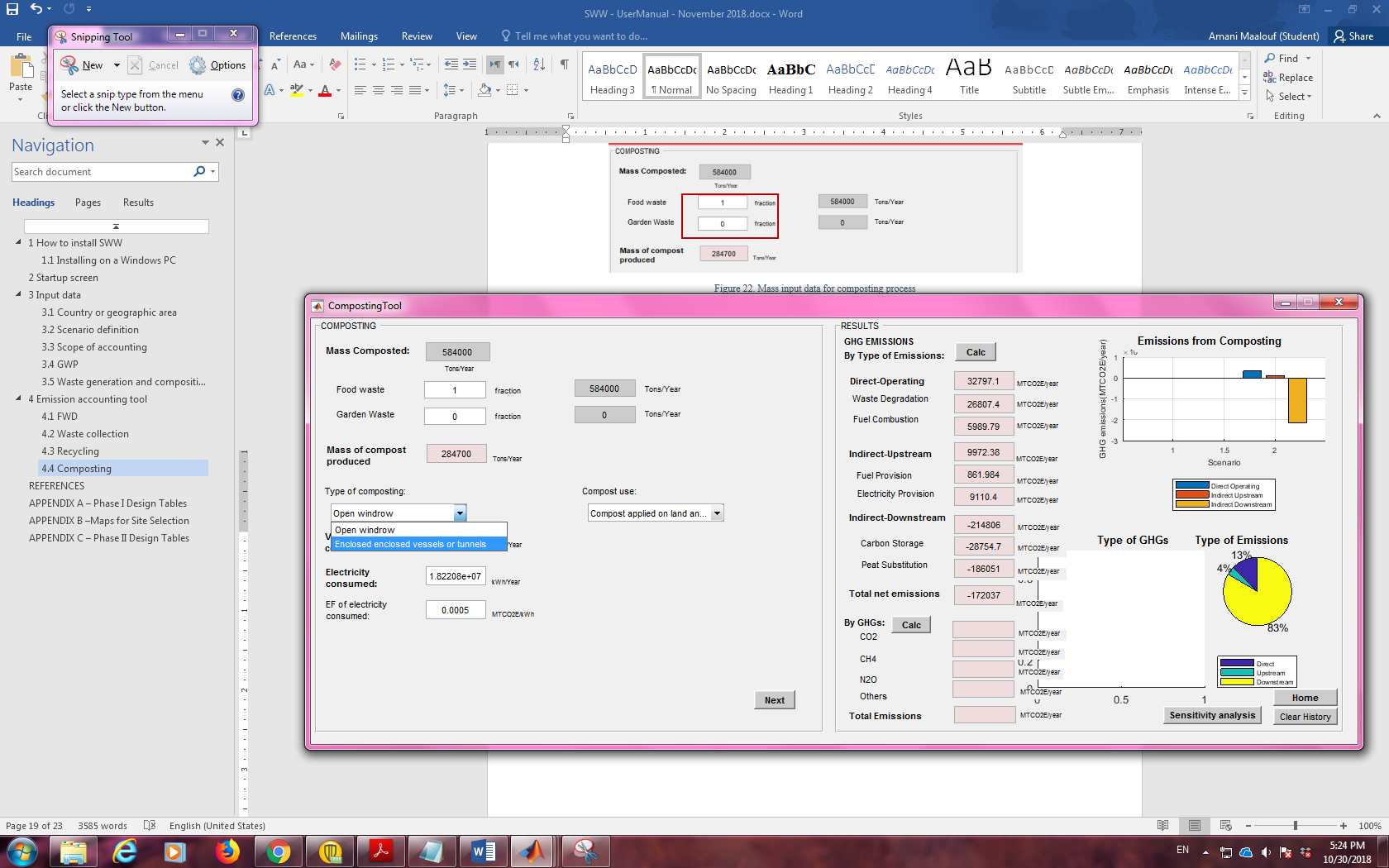 |
| --- | --- |

Figure SM 16. Select composting technology

Emissions from composting process are disaggregated by type (direct or indirect) and by gas as shown in Figure SM 17 and Figure SM 18. Direct GHG emissions from waste decomposition during composting consist of biogenic CO_2_ (considered neutral), N_2_O, and minor amounts of CH_4_, as well as emissions from fuel combustion by on-site mobile equipment. Indirect emissions from composting include upstream emissions from electricity consumption and the provision of fuel with corresponding emission factor related to extraction, processing, storage, and transportation of the fuel. Indirect downstream emissions consist of avoided emissions from carbon storage associated with the application of compost to soils or substitution of peat production. The latter is selected from the drop-down menu of “compost use” (see Figure SM 16).


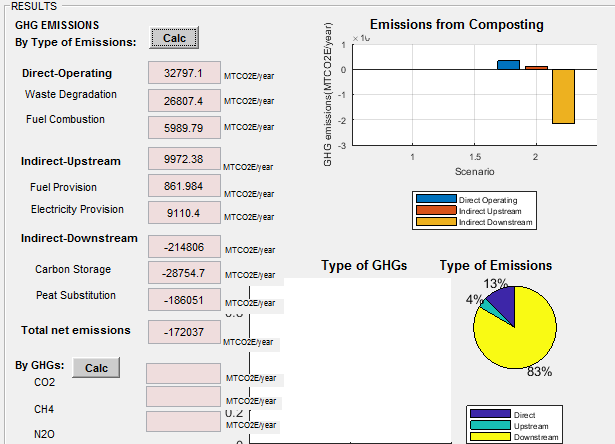


Figure SM 17. Net total emissions from composting process disaggregated by type


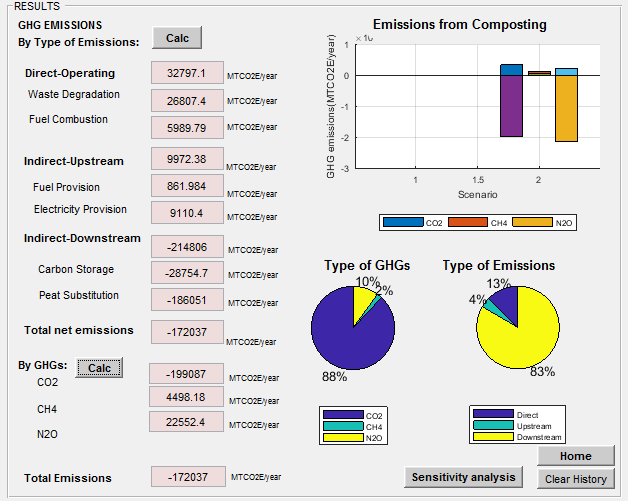


Figure SM 18. Net total emissions from composting process calculated by gas

The software allows the user to select key parameters for sensitivity and uncertainty assessment whereby each parameter can be individually modified to assess its impact on emissions by following two methods: 1) the One-at-a-time (OAT) analysis or 2) Monte Carlo analysis. When running a single case scenario, the user has the option to select parameters for uncertainty assessment by clicking on “Sensitivity analysis” as shown in Figure SM 18 with box marked in red. Parameters are in this case scenario specific, so they are used to assess a specific process. After you select the parameter, calculate the emissions again by clicking on “Calc”. Results will be displayed in the sensitivity analysis window after you run the model from the main window in Figure M1.

## Anaerobic digestion

The software keeps track automatically of all the mass and material flows specific for each process as displayed in Figure SM 19. The mass of waste anaerobically digested is based on the fraction of MSW that is sent for anaerobic digestion as selected by the user in the main window (“3” in Figure M1) The user must input the fractions of waste materials composted (marked with a dashed red box in Figure SM 19). In case the fraction of waste sent for anaerobic digestion are left empty, by default, SWW considers that food waste constitutes 100% of waste anaerobically digested. SWW will then automatically calculates the total mass anaerobically digested, waste materials anaerobically digested, and mass of compost produced Figure SM 19.


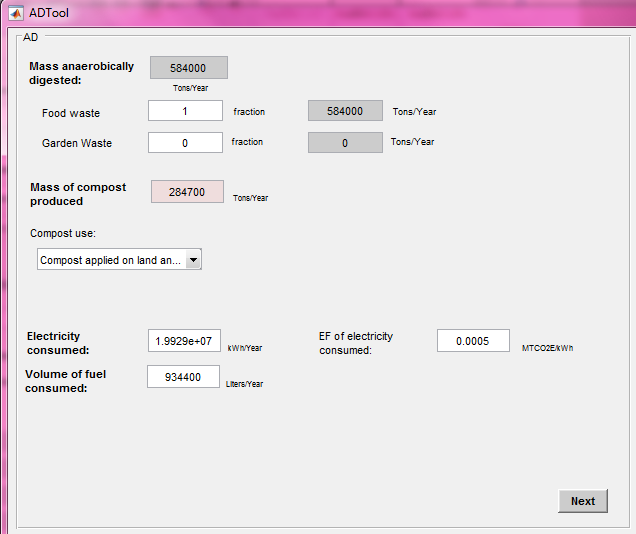


Figure SM 19. Data input for anaerobic digestion process

The user must then input the amount of fuel and electricity consumed for the operation of the anaerobic digestion process. In case these values are left empty, SWW calculates them based on default data. After filling all required input data click on “Next” button to display all values and proceeds to calculate net total emissions.

Emissions from anaerobic digestion process are disaggregated by type (direct or indirect) and by gas as shown in Figure SM 20 and Figure SM 21. Net emissions from anaerobic digestion include direct emissions from fugitive CH_4_ during waste degradation and emissions from fuel consumption of onsite operating equipment. Indirect emissions include upstream emissions from electricity and fuel provision. Indirect downstream or avoided emissions from carbon storage are associated with the electricity production from biogas collected as well as application of compost to soils or substitution of peat production. The latter is selected from the drop-down menu of “compost use” (marked with a red box in Figure SM 19).


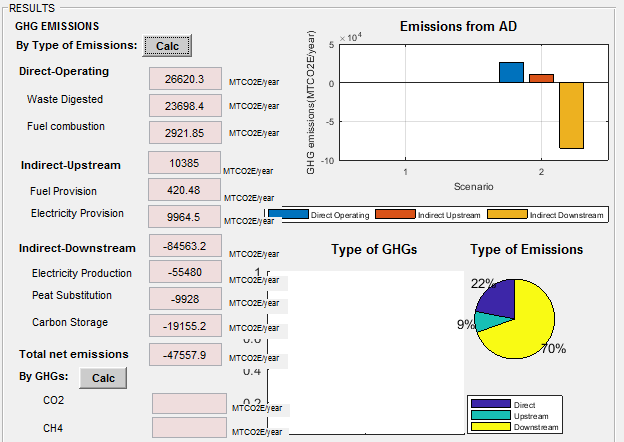


Figure SM 20. Net total emissions from anaerobic digestion process disaggregated by type


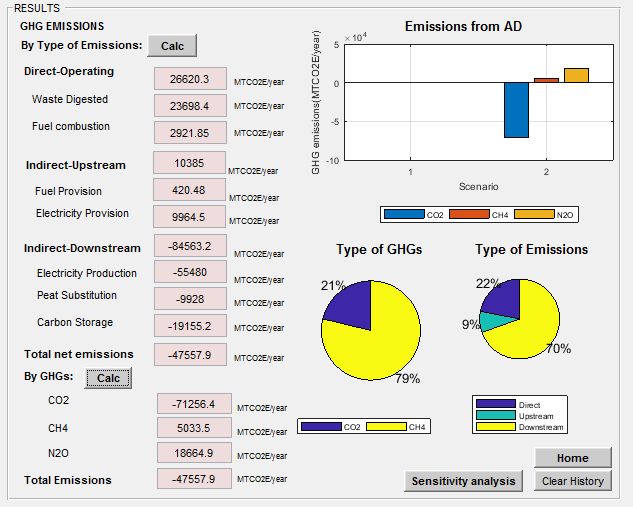


Figure SM 21. Net total emissions from anaerobic digestion process disaggregated by gas

## Waste combustion

The software keeps track automatically of all the mass and material flows specific for each process as displayed in Figure SM 22. The mass of waste incinerated is based on the fraction of MSW that is sent for incineration as selected by the user in the main window (“3” in Figure M1). SWW automatically calculates the mass of waste combusted as displayed in Figure SM 22.


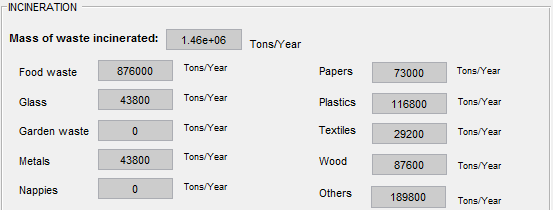


Figure SM 22. Mass input data for incineration process

The user must select from the combustion system (e.g. incineration with energy recovery, without energy recovery, or open burning) from the drop-down menu in Figure SM 23.

In case of selecting the incineration process, the user must input the amount of fuel necessary for the operation process, the combustion system efficiency, the amount of electricity consumed, the waste calorific value, the amount of electricity produced, the type of auxiliary fuel used to supply the combustion process (which is selected from the drop-down menu) and the amount of bottom ash produced with corresponding management process (selected from the drop-down menu) Figure SM 23. In case these values are left empty, SWW provides default averages reported in the literatures and displays the values after clicking on the “Next” button Figure SM 23.

| 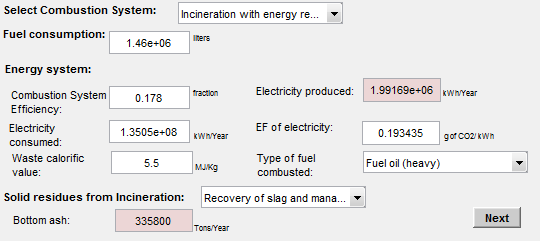 | 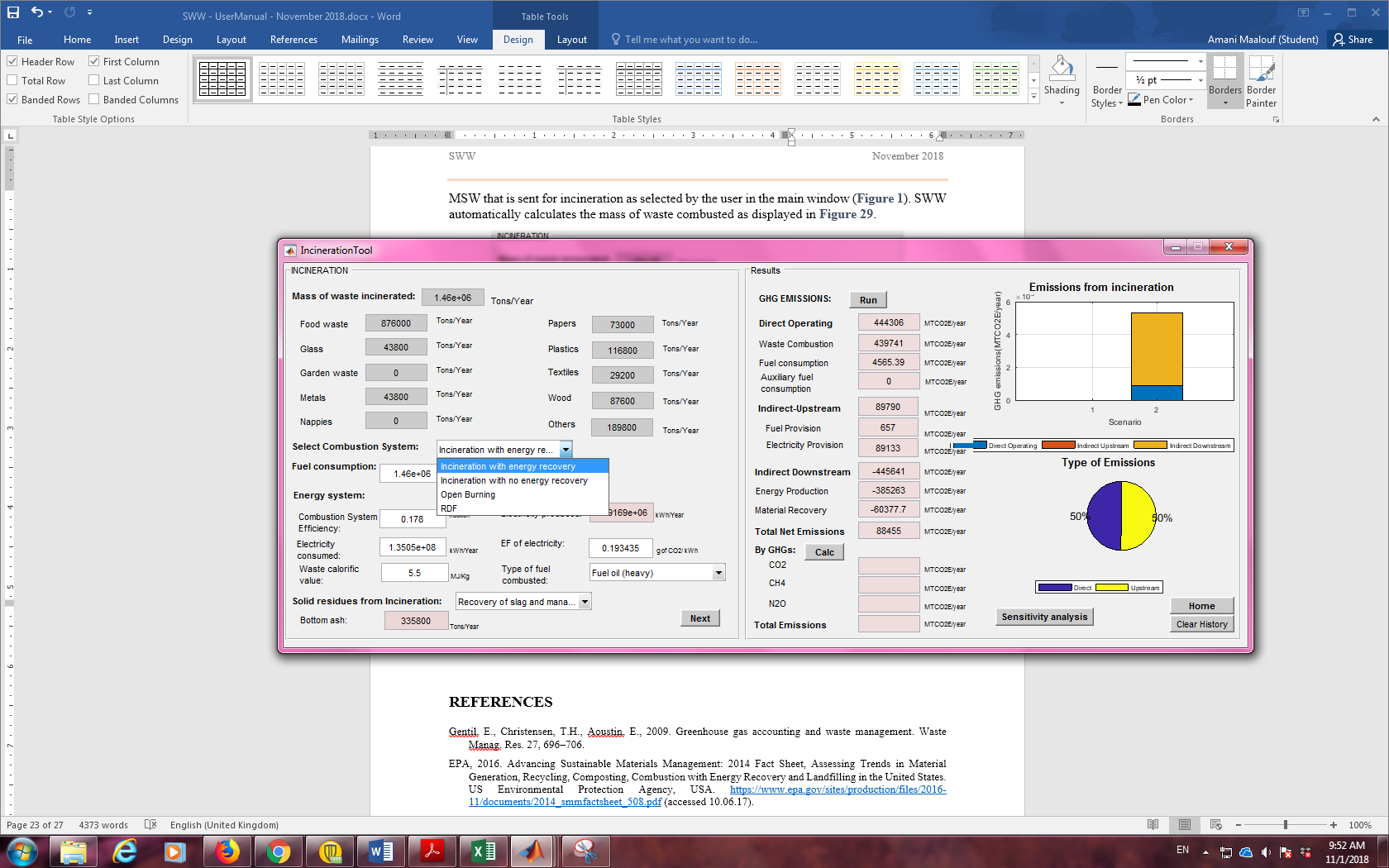 |
| --- | --- |

Figure SM 23. Data input for the combustion process

Emissions from the incineration process are disaggregated by type (direct or indirect) and by gas as shown in Figures SM 24 and SM 25. Direct emissions from incineration of individual waste material as well as emissions from fuel combustion of onsite operating equipment. The latter also includes the auxiliary amount of fuel needed when the low heating value (LHV) of waste is less than 5–6 MJ/kg, which is required to sustain the burning process. This is particularly important in developing economies where waste is characterized by a high biodegradable organic fraction and a high moisture content leading to a lower calorific value. SWW accounts for a wide range of emission factors associated with various types of auxiliary fuel (e.g. diesel/gas oil, fuel oil, and hard coal for power plants) to satisfy the LHV depending on the study area (country or region) with averages adopted from [3].

Indirect emissions include emissions from the management of solid residues generated from waste incineration (savings from slag recovery and load from bottom ash landfilling) as well as avoided emissions from electricity production which depends on the:

- - 1. Energy content of mixed waste or of waste components combusted in kWh/tonne of waste,
    2. Combustion system efficiency in converting the energy content of waste materials to recovered electricity, and
    3. The emission factor of electricity avoided.

SWW also accounts for direct emissions during waste combustion from open burning, which is a common practice in developing economies.


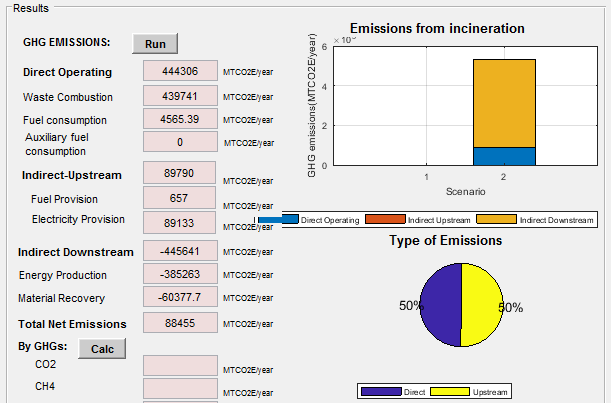


Figure SM 24. Net total emissions from incineration process disaggregated by type


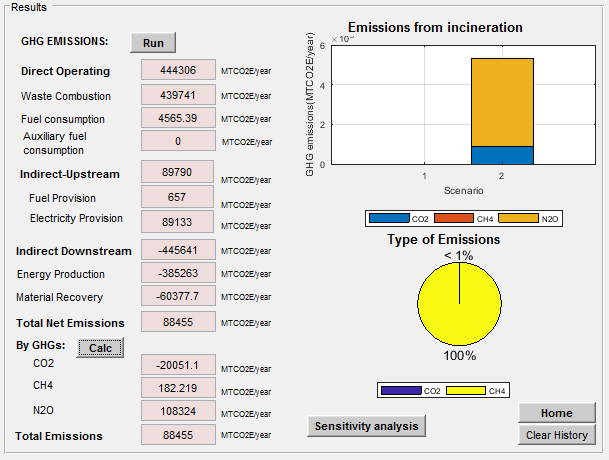


Figure SM 25. Net total emissions from the incineration process disaggregated by gas

## Landfilling

The software keeps track of the mass and material flows specific to each process as displayed in Figure SM 26. The mass of waste landfilled is based on the fraction of MSW that is sent for landfilling as selected by the user in the main window (“3” in Figure M1). SWW automatically calculates the mass of waste landfilled as marked in the dashed red box in Figure SM 26. The user must then input the data related to the landfilling process. If these values are left empty, SWW provides default averages adopted from the literature (refer to [10] for further details).

Emissions from landfilling process are disaggregated by type (direct or indirect) and by gas as shown in Figures SM *27* and SM 28. Direct emissions from landfilling consist of emissions from waste degradation (CH_4_ and N_2_O) and fuel used by onsite activities (mobile equipment, electric generators, dozers, compactors and other landfill vehicles). SWW estimates emissions from landfills 1) without landfill gas (LFG) recovery systems; (2) with flaring of recovered CH_4_ and N_2_O emissions; (3) combustion of CH_4_ for energy recovery, depending on the LFG recovery scenario. The latter can be selected by the user from the drop-down menu (marked with the red box) in Figure SM 26. Note that CO_2_ emissions from waste degradation and flaring are considered as biogenic sources. SWW allows the estimation of methane emissions from individual waste components in accordance to the theoretical yield assuming that CH_4_ emissions are released in the same year of waste deposition. Fuel combusted by waste collection trucks while unloading at and driving to the site are ascribed to collection and transport of waste.


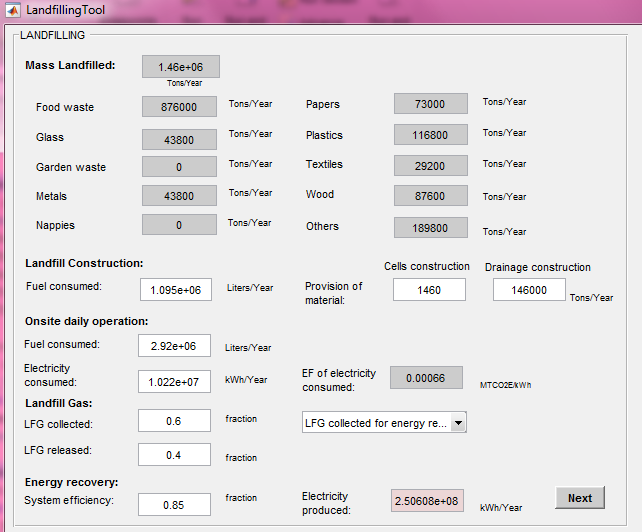


Figure SM 26. Data input for the landfilling process


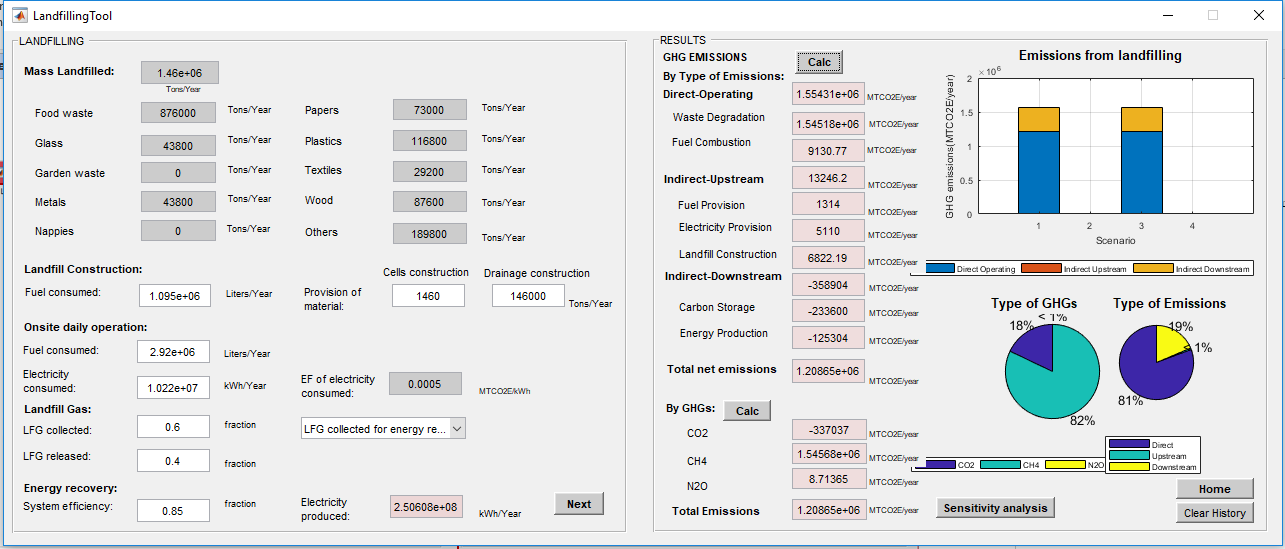


Figure SM 27. Net total emissions from landfilling disaggregated by type


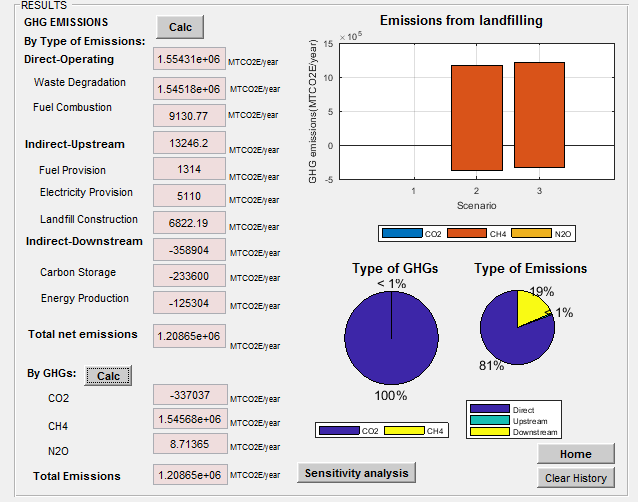


Figure SM 28.Net total emissions from landfilling disaggregated by gas

Indirect emissions from landfilling of waste include upstream emissions associated with electricity and fuel provision as well as emissions during landfill construction (provision of liner materials and construction of drainage system, etc.).

Indirect downstream emissions consist of avoided emissions from carbon storage and electricity production, which is dependent on the (1) energy content of recovered methane being combusted in kWh/MT of CH_4_ recovered; (2) capacity factor for electricity generation, a; and (3) the emission factor of electricity avoided, depending on the energy mix in the study area (country or region).

Future work will expand the emission accounting from the landfilling process to account for leachate occurrence and minimization as well as optimize the landfill site selection process [4,5].

## Open dumping

The software keeps track of the mass and material flows specific for each process as displayed in Figure SM 29. The mass of waste that is open dumped is based on the fraction of MSW that is diverted to open dumping as selected by the user in the main window (“3” in Figure M1). SWW calculates the mass of waste open dumped as marked in the dashed red box in Figure SM 29. Open dumping comprises sites not meeting the criteria of managed solid waste disposal sites and which are classified as shallow (depth < 5 m) or deep (depth > 5m and/or high-water table near ground level) [6]. The latter is selected from the drop-down menu marked in the dashed red box in Figure SM 29.


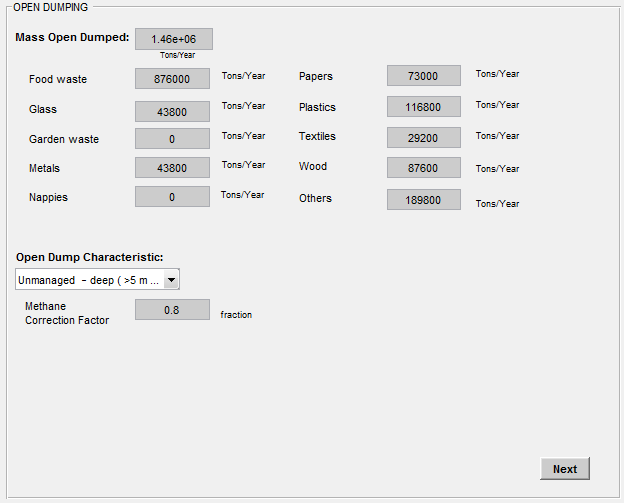


Figure SM 29. Data input for open dumping of waste

Emissions from open dumping are disaggregated by type (direct or indirect) as shown in Figure SM 30. Direct emissions from open dumping of waste are calculated relative to controlled landfill sites whereby a methane correction factor is used [6] to account for a larger fraction of waste that is likely to decompose aerobically contributing to a lower amount of methane generation.


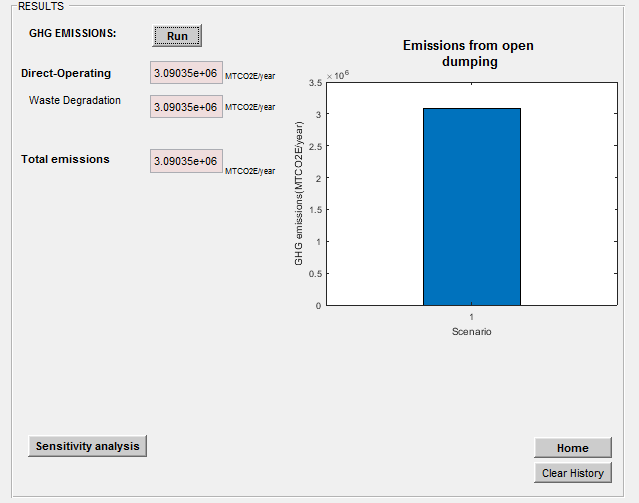


Figure SM 30. Total emissions from open dumping

# REFERENCES

[1] N. Marashlian, M. El-Fadel, The effect of food waste disposers on municipal waste and wastewater management. Waste Manage. Res. (2005) 23, 20-31.

[2] EPA/ICF, Documentation for Greenhouse Gas Emission and Energy Factors Used in the Waste Reduction Model (WARM): Background Chapters (WARM V.14), U.S. EPA Office of Resource Conservation and Recovery, Washington DC, 2016.

[3] T. Fruergaard, T. Astrup, T. Ekvall, Energy use and recovery in waste management and implications for accounting of greenhouse gases and global warming contributions. Waste Manage. Res. (2009) 27, 724-737.

[4] H. Luo, Y. Chenga, D. Hea, E-H. Yang, Review of leaching behavior of municipal solid waste incineration (MSWI) ash. Sci. Total Environ. Sci. (2019) 668, 90-103.

[5] H. Luo, Y. Zeng, Y. Cheng, D. He, X. Pan, Recent advances in municipal landfill leachate: A review focusing on its characteristics, treatment, and toxicity assessment. Sci. Total Environ. Sci. (2020) 703, 135468.

[6] IPCC, Guidelines for National Greenhouse Gas Inventories, in: H.S. Eggleston, L. Buendia, K. Miwa, T. Ngara, K. Tanabe, (Eds.), National Greenhouse Gas Inventories Programme, IGES, Japan, 2006.
